# Phosphorylation of chemoattractant receptors regulates chemotaxis, actin reorganization and signal relay

**DOI:** 10.1242/jcs.122952

**Published:** 2013-10-15

**Authors:** Joseph A. Brzostowski, Satoshi Sawai, Orr Rozov, Xin-hua Liao, Daisuke Imoto, Carole A. Parent, Alan R. Kimmel

**Affiliations:** 1 Laboratory of Immunogenetics Imaging Facility, NIAID/NIH, Rockville, MD 20852, USA; 2 Graduate School of Arts and Sciences, University of Tokyo and PRESTO, JST, Tokyo 153-8902, Japan; 3 Laboratory of Cellular and Developmental Biology, NIDDK/NIH, Bethesda, MD 20892, USA; 4 Graduate School of Arts and Sciences, University of Tokyo, Tokyo 153-8902, Japan; 5 Laboratory of Cellular and Molecular Biology, NCI/NIH, Bethesda, MD 20892, USA

**Keywords:** GPCR, cAMP, PI3K, TORC2, AKT, Oscillations, Heterotrimeric G proteins, ERK2

## Abstract

Migratory cells, including mammalian leukocytes and *Dictyostelium*, use G-protein-coupled receptor (GPCR) signaling to regulate MAPK/ERK, PI3K, TORC2/AKT, adenylyl cyclase and actin polymerization, which collectively direct chemotaxis. Upon ligand binding, mammalian GPCRs are phosphorylated at cytoplasmic residues, uncoupling G-protein pathways, but activating other pathways. However, connections between GPCR phosphorylation and chemotaxis are unclear. In developing *Dictyostelium*, secreted cAMP serves as a chemoattractant, with extracellular cAMP propagated as oscillating waves to ensure directional migratory signals. cAMP oscillations derive from transient excitatory responses of adenylyl cyclase, which then rapidly adapts. We have studied chemotactic signaling in *Dictyostelium* that express non-phosphorylatable cAMP receptors and show through chemotaxis modeling, single-cell FRET imaging, pure and chimeric population wavelet quantification, biochemical analyses and TIRF microscopy, that receptor phosphorylation is required to regulate adenylyl cyclase adaptation, long-range oscillatory cAMP wave production and cytoskeletal actin response. Phosphorylation defects thus promote hyperactive actin polymerization at the cell periphery, misdirected pseudopodia and the loss of directional chemotaxis. Our data indicate that chemoattractant receptor phosphorylation is required to co-regulate essential pathways for migratory cell polarization and chemotaxis. Our results significantly extend the understanding of the function of GPCR phosphorylation, providing strong evidence that this evolutionarily conserved mechanism is required in a signal attenuation pathway that is necessary to maintain persistent directional movement of *Dictyostelium*, neutrophils and other migratory cells.

## Introduction

The ability of eukaryotic cells to migrate directionally within extracellular chemoattractant gradients is essential for numerous biological processes. Most chemoattractant receptors are members of the G-protein-coupled receptor (GPCR) superfamily, which integrates heterotrimeric G-protein signaling to direct downstream effector networks. Many processes that regulate receptor-mediated chemotactic movement are remarkably conserved through evolution. Such mechanistic universality and ease of experimental manipulation have made *Dictyostelium* a premier system for the discovery and analyses of regulatory signaling networks that are common to most migratory cells, including human neutrophils and macrophages (Jin et al., 2009).

As a population of *Dictyostelium* depletes nutrients within its local environment, starved cells enter a cooperative developmental program leading to multicellular aggregation (McMains et al., 2008; Schaap, 2011). Upon nutrient depletion, cells secrete cAMP, which acts as the extracellular chemoattractant to coordinate directed cell movement. Synthesis, secretion and degradation of cAMP are temporally and spatially organized, ensuring a periodic release of cAMP from initiating signaling centers (McMains et al., 2008; King and Insall, 2009; Swaney et al., 2010); neighboring cells simultaneously relay the cAMP signal outwardly and move inwardly, towards the centers of cAMP production. The response networks that promote cAMP relay and chemotactic movement are transiently activated upon stimulation. Following adaptation (desensitization) to the chemoattractant signal, cAMP synthesis is suppressed and extracellular cAMP signals are degraded by a secreted phosphodiesterase (PDE). Adapted cells remain transiently refractory to additional stimulation until they de-adapt (resensitize) for another round of cAMP signal relay and movement.

*Dictyostelium* detect cAMP through surface cAMP receptors (CARs), which in turn, activate multiple downstream pathways through heterotrimeric G proteins (McMains et al., 2008; King and Insall, 2009; Swaney et al., 2010). The aggregation-specific, cAMP-generating enzyme adenylyl cyclase ACA is activated by a rise in receptor occupancy, but activation is transient. If a continuous cAMP stimulus is applied, the ACA response remains adapted. Other downstream pathways in *Dictyostelium* also exhibit adaptive/de-adaptive regulation, including Ras-GTP cycling, phosphatidylinositol (3,4,5)-trisphosphate (PIP_3_) and cGMP production, actin polymerization and various kinase activities (Futrelle et al., 1982; McMains et al., 2008; King and Insall, 2009; Swaney et al., 2010). However, only few molecular components have been identified in *Dictyostelium* that regulate adaptive responses, and none seem to act on all targets (Brzostowski et al., 2004). Indeed, the temporal kinetics of the different adaptive responses is sufficiently disparate that multiple pathways could impact adaptation. Further, adaptation must function independently of ligand-stimulated dissociation of Gα–βγ, because these complexes remain constitutively disassociated during adaptation in the presence of saturating levels of cAMP (Janetopoulos et al., 2001).

It is well established in mammalian cells that ligand-induced phosphorylation of GPCRs will recruit arrestin family proteins, which uncouple receptors from downstream G proteins (Pitcher et al., 1998; Ferguson, 2001; Shenoy and Lefkowitz, 2011; Shukla et al., 2011; Evron et al., 2012). Arrestin binding also promotes receptor internalization and downregulation of ligand detection and occupancy, while simultaneously activating a series of G-protein-independent events. Similar to GPCRs in mammalian cells, CAR1 is phosphorylated at multiple cytoplasmic residues upon chemoattractant stimulation (Hereld et al., 1994). CAR1 phosphorylation/dephosphorylation oscillates concomitantly with the periodic rise and fall of extracellular cAMP during aggregation (Klein et al., 1985), yet CAR1 phosphorylation is non-adaptive and persists if cAMP concentrations are constant (Vaughan and Devreotes, 1988). Receptor downregulation in *Dictyostelium*, as defined by the loss of cAMP binding sites (Van Haastert, 1987a) or receptor internalization (Sergé et al., 2011), is observed in cells exposed to saturating (µM) levels of ligand. At sub-saturating, non-varying cAMP concentrations, which promotes adaptation/desensitization, binding sites are maintained, but with modified affinity (Van Haastert, 1987a). Despite the strong correlation between the rise and fall of CAR1 phosphorylation and adaptive kinetics of certain downstream pathways, earlier studies had suggested that receptor phosphorylation in *Dictyostelium* might not attenuate G-protein signaling as it does in mammalian cells (Hereld et al., 1994; Kim et al., 1997; Briscoe et al., 2001). However, these studies had been limited by the assays available at that time and did not fully exclude a role for receptor phosphorylation during chemotactic signaling. Indeed, cells expressing phosphorylated or non-phosphorylated CAR1 did not respond to cAMP identically (Hereld et al., 1994; Kim et al., 1997; Briscoe et al., 2001). Cells expressing non-phosphorylated CAR1 had an altered F-actin pattern and reduced response to cAMP in two-drop assays. Aberrant cAMP wave propagation was noted, but was not analyzed further. Thus, there was a possible conundrum for the functional consequence of receptor phosphorylation regarding chemotaxis in *Dictyostelium*.

Recent studies in mammalian cells have suggested a GPCR- and arrestin-dependent linkage to actin re-organization and chemotactic migration (DeFea, 2007; Min and Defea, 2011). Because the function of CAR1 phosphorylation during chemotaxis in *Dictyostelium* had not been fully addressed (Hereld et al., 1994; Kim et al., 1997; Briscoe et al., 2001), we have chosen to re-investigate its role during cell movement, focusing on biological processes and technologies that were previously unavailable for analyses. Remarkably, we find that loss of CAR1 phosphorylation has a considerable negative impact on persistent directional movement with a major defect in the regulation of protracted F-actin polymerization. Additionally, we show that long-range extracellular cAMP signal relay is abrogated in cells lacking CAR1 phosphorylation. This results from disruption of ACA adaptation, but is independent of adaptation of the ACA activators TORC2 and PI3K. Our data now show that multiple, but not all, signaling pathways are impacted in *Dictyostelium* upon disruption of CAR1 phosphorylation, indicating that CAR1 phosphorylation, indeed, models mammalian GPCRs and suggests that chemotaxis in mammalian cells might be affected in mechanistically similar manners.

## Results

### Receptor phosphorylation regulates chemotaxis

To evaluate the role of CAR1 phosphorylation during chemotaxis, we compared the functions of wild-type CAR1 (WT-CAR1) and a non-phosphorylatable CAR1 mutant (CM1234), where all C-terminal serine residues were removed by either deletion or substitution with alanine or glycine (Hereld et al., 1994). These CAR1 variants were constitutively expressed at comparable levels in a strain (*car1*^−^/*car3*^−^) that lacks both CAR1 and CAR3 (supplementary material Fig. S1) (Caterina et al., 1994), the cAMP receptors that mediate early chemotactic response during *Dictyostelium* development (Saxe et al., 1991; Insall et al., 1994b). Expression of WT-CAR1 fully rescued the *car1*^−^/*car3*^−^ phenotype (see below) (Kim et al., 1997).

**Fig. 1.**
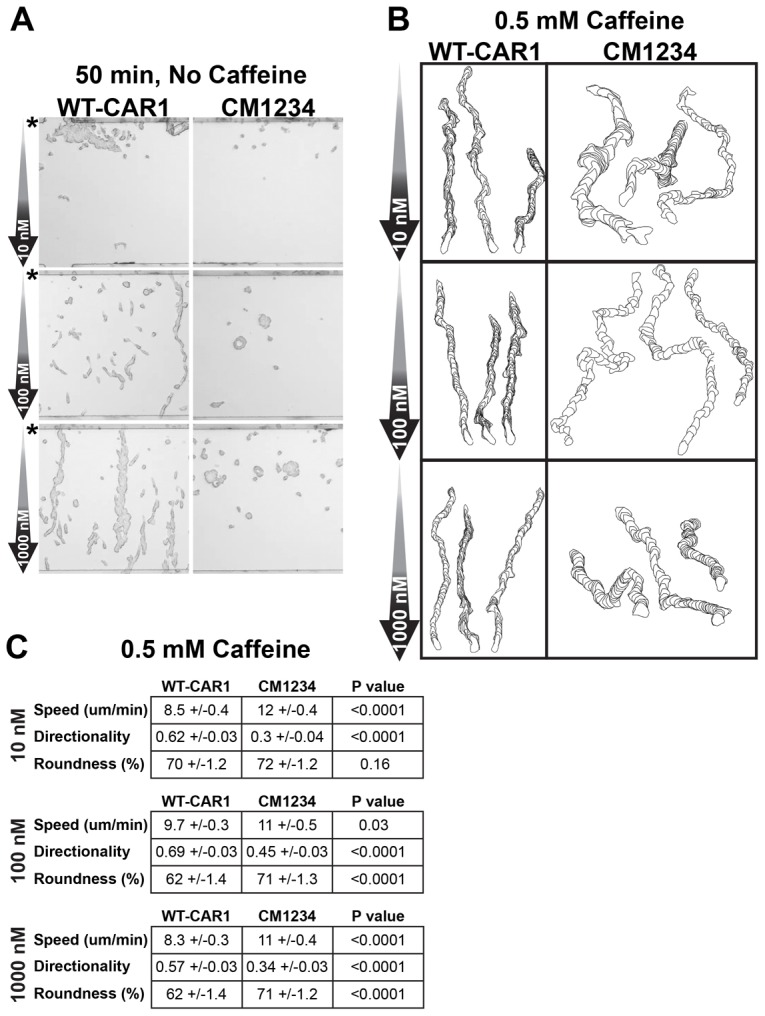
**Loss of CAR1 phosphorylation impairs directional migration within a linear cAMP gradient.** (A) Differentiated WT-CAR1 or CM1234 cells were exposed to linear cAMP gradients, as indicated, in an EZ-TAXIScan chamber and assayed over time for directional movement. Origin of cells in chamber is indicated (*). The 50 minute time point is shown. See supplementary material Fig. S2 for additional time points and supplementary material Movie 1 for an image sequence. (B) Differentiated WT-CAR1 or CM1234 cells were assayed as in Fig. 1A, but in the presence of 0.5 mM caffeine. cAMP concentrations used to initiate the gradient are indicated. See supplementary material Movie 2 for an image sequence. Shown are single-cell-outline traces using the DIAS software package. The cell tracings were stacked for 50 frames and compiled into one image to display morphometrics of the sequence. (C) Metrics were determined from tracings of ∼100 caffeine-treated cells in Fig. 1B using DIAS (see supplementary material Movie 2). Directionality (the net path length divided by the total path length) was measured using the chamber bottom as the reference point; a value of 1.0 corresponds to a completely straight path, whereas smaller values indicate a meandering path. 100% roundness indicates a circular cell, whereas 0% corresponds to a cell having no effective width or enclosed measurable area. *P* values of Student *t*-tests are shown.

WT-CAR1- and CM1234-expressing cells were differentiated in culture to the chemotactically competent stage and chemotaxis was assayed in linear cAMP gradients using the EZ-TAXIScan chamber (Nitta et al., 2007). WT-CAR1 cells migrated directionally toward the gradient source at each cAMP concentration tested (Fig. 1A; supplementary material Fig. S2, Movie 1), but also formed long cell-to-cell streams as a result of the secretion of endogenous cAMP and chemotactic movement toward the cAMP-secreting cells. In comparison, the directional chemotaxis of CM1234 cells within the exogenous cAMP gradient was extremely poor. At the lowest cAMP concentration tested (10 nM; Fig. 1A; supplementary material Fig. S2, Movie 1), CM1234 cells did not exit the starting chamber; at higher cAMP concentrations, cells followed meandering paths and coalesced into aggregates at the center of the chamber, suggesting that endogenous cell-to-cell cAMP signaling overwhelmed the external cAMP gradient (Fig. 1A; supplementary material Fig. S2, Movie 1).

To analyze chemotactic responses solely dependent upon the application of exogenous cAMP, cells were assayed in the presence of varying concentrations (0.5–2 mM) of caffeine, which inhibits endogenous cAMP production in *Dictyostelium* (Brenner and Thoms, 1984). In the presence of caffeine, WT-CAR1 cells did not form streams, but migrated individually and directionally toward the gradient source with metrics similar to those of other wild-type strains (Fig. 1B,C; supplementary material Movie 2). Caffeine also inhibited the formation of CM1234 cell aggregates, enabling measurement of cell behaviors to the applied cAMP. Data from cell traces show that CM1234 cells migrated markedly faster than WT-CAR1 cells, and turned more frequently, with less directionality toward the exogenous cAMP (Fig. 1C). Under all caffeine and cAMP gradient conditions (supplementary material Movies 2 and 3), CM1234 cells displayed enhanced turning, hyperactive pseudopod extension and retraction, and poor elongation compared with WT-CAR1 cells (Fig. 1C; supplementary material Movies 4 and 5). Similar chemotactic defects were observed for CM1234 cells in the absence of caffeine, as assayed in EZ-TAXIScan chambers at low cell density to minimize cell–cell interactions (supplementary material Fig. S3, Movies 6 and 7). We conclude that cells that express a non-phosphorylatable variant of CAR1 have impaired chemotaxis and might exhibit enhanced cAMP signaling.

### Long-range cAMP wave propagation is defective in CM1234 cells

The propensity of CM1234 cells to aggregate even in the presence of a strong, exogenous cAMP gradient (see Fig. 1A; 1000 nM cAMP) suggests that loss of receptor phosphorylation leads to enhanced cAMP signaling. To determine whether phosphorylation-deficient receptors alter extracellular cAMP relay, we used dark-field, time-lapse microscopy to image cAMP wave propagation during aggregation.

**Fig. 2.**
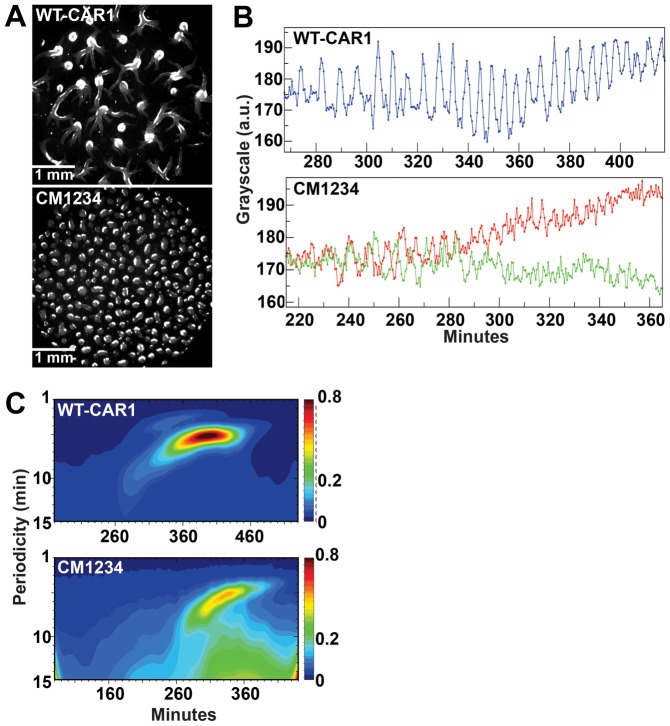
**Loss of CAR1 phosphorylation impairs long-range cAMP signal relay.** (A) WT-CAR1 and CM1234 cells were starved on non-nutrient agar surfaces and oscillatory cell shape changes imaged by dark-field microscopy and digitally recorded. Each frame is subtracted pixel by pixel from the subsequent frame to create a frame-subtracted image sequence for higher contrast. The single dark-field images shown are the mound phenotypes for the WT-CAR1 and CM1234 cells. Outwardly moving cAMP gradients are generated periodically from regularly spaced signaling centers that arise during development and are visualized under low magnification as optical density waves of alternating bands of bright migrating cells and dark bands of randomly oriented, quiescent cells (Alcantara and Monk, 1974; Tomchik and Devreotes, 1981; Sawai et al., 2007). See supplementary material Movies 8 and 9 for an example of a dark-field and frame-subtracted sequence for both WT-CAR1 and CM1234 cells. (B) Time-plots were obtained by measuring intensity changes (a.u. = arbitrary units) in an arbitrary region through the frame-subtracted image sequence. WT-CAR1 cells produce robust oscillations with a ∼5 minute period that are representative of all measurements in an aggregation field. Two measurements for CM1234 cells are shown to demonstrate their long-range signal disorganization. (C) Shown are wavelet contour plots of cellular oscillations through the frame-subtracted image sequences. The periodicity (minutes, *y*-axis) of synchronized oscillations over the time course (minutes, *x*-axis) yields the peak wavelet amplitude W(s;t) (for color scale, see Sawai et al., 2005).

WT-CAR1 and CM1234 cells were washed from growth medium and plated on solid substrata in non-nutrient developmental buffer. WT-CAR1 and CM1234 cells were recorded during autonomous cAMP signaling and aggregate formation, and the images digitally analyzed (the aggregate mound stage is shown in Fig. 2A and the corresponding time-lapse sequences in supplementary material Movies 8 and 9). WT-CAR1 cells displayed characteristic properties of cell-to-cell streaming during aggregate formation. By contrast, cell-to-cell streaming was not observed for the CM1234 cells, but, as CM1234 cells establish many more aggregation territories over smaller areas, aggregation occurred more quickly in CM1234 cells relative to WT-CAR1, as noted previously (Hereld et al., 1994; Kim et al., 1997; Briscoe et al., 2001).

Amplitude, periodicity, onset and cessation of optical density oscillations (Gross et al., 1976; Siegert and Weijer, 1989; Sawai et al., 2005) were determined by monitoring the change in grayscale values in subregions of frame-subtracted images (Fig. 2B) and by wavelet analyses (Sawai et al., 2005; Sawai et al., 2007) during the entire image time sequence (Fig. 2C). The periodic changes in optical density mainly reflect cell-shape changes in response to the passage of chemoattractant waves (Kamino et al., 2011). WT-CAR1 cells produced robust oscillations and propagated long-range optical density waves with ∼5 minute periodicities, which were coordinated through the majority of the cell population (Fig. 2B,C; supplementary material Movies 8 and 9). WT-CAR1 oscillations initiated at ∼270 minutes post-starvation, peaked at ∼400 minutes, and persisted for an additional ∼60 minutes (Fig. 2B; supplementary material Movies 8 and 9). CM1234 cells also produced oscillations with a ∼5-minute period, however, the oscillations were detected ∼1 hour earlier than in WT-CAR1 cells and had much weaker amplitudes and more asynchronous patterns (Fig. 2B). In addition, the oscillations persisted for much shorter intervals (∼1 hour; Fig. 2C) and with significantly suppressed long-range responses (Fig. 2A; supplementary material Movies 8 and 9). Together these findings suggest that enhanced cAMP signaling in CM1234 cells disrupts cell streaming and aggregation formation.

**Fig. 3.**
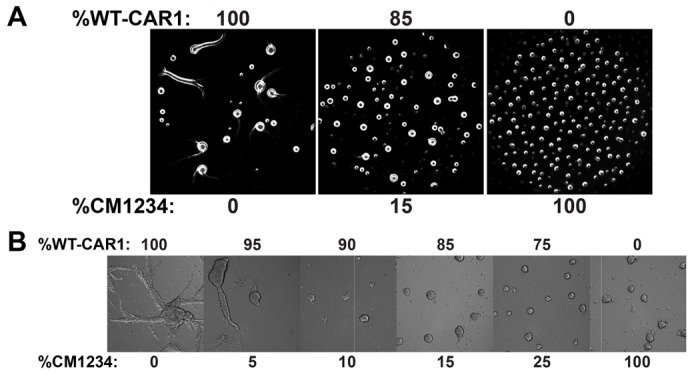
**Chimeric development of WT-CAR1 and CM1234 cells.** (A) WT-CAR1 and CM1234 cells were mixed at the indicated ratios, plated for development on non-nutrient agarose and imaged by time-lapse microscopy on a motorized stage. Images shown are the mound phenotypes. See supplementary material Movie 10 to compare oscillations in frame-subtracted sequences among pure populations and chimeric mixed cells. (B) WT-CAR1 and CM1234 cells were mixed at the indicated ratios, plated for development on plastic surfaces submerged under DB in a multiwell plate, and imaged by time-lapse microscopy on a motorized stage. Images shown reflect the steaming capacity in the various mixes. See supplementary material Movie 11 for examples of two time sequences.

If CM1234 cells exhibit enhanced cAMP signaling, they would be predicted to alter the phenotype of WT cells during chimeric development (Brzostowski et al., 2002). We tested this by mixing WT-CAR1 and CM1234 cells at different ratios and recorded pure-population and chimeric development by dark-field, time-lapse microscopy. We found that chimeras of 85% WT-CAR1 and 15% CM1234 cells had disrupted signal-relay and decreased mound size, closely mimicking a population of 100% CM1234 cells (Fig. 3A; supplementary material Movie 10). We also examined the development of chimeras in under-buffer assays. Here, phenotypic differences between populations of 100% WT-CAR1 and 100% CM1234 cells were also apparent (Fig. 3B; supplementary material Movie 11). Pure WT-CAR1 populations formed large streaming territories, whereas 100% CM1234 cells formed small aggregates with minimal streaming. When chimeras were analyzed, WT-CAR1 streaming was blocked with as few as 10% CM1234 cells and these chimeric mounds were nearly indistinguishable from a 100% CM1234 cell population (Fig. 3B; supplementary material Movie 11). These findings suggest that the chimeric phenotype is non-cell autonomous and is probably due to the overproduction of secreted cAMP by CM1234 cells.

### Receptor phosphorylation regulates ACA adaptation

**Fig. 4.**
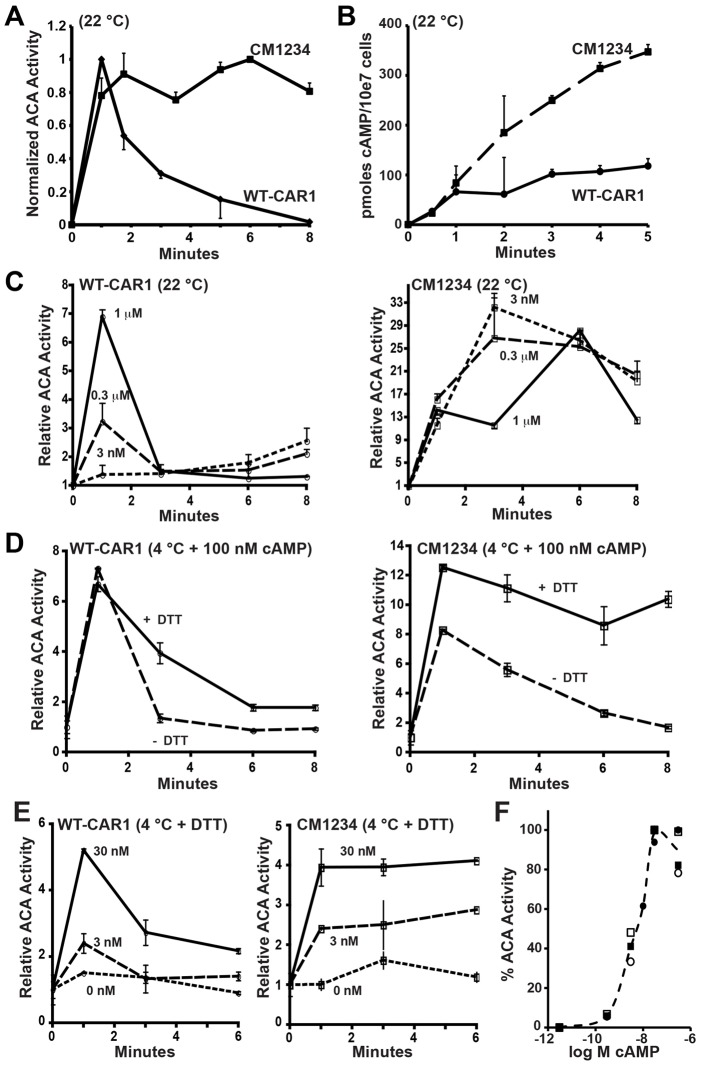
**Non-adaptive response of ACA in CM1234 cells.** (A) Differentiated WT-CAR1 (*n* = 5) and CM1234 (*n* = 5) cells were stimulated at 22°C with 10 µM cAMP. At the times indicated, lysates were prepared and ACA activity measured for 1 minute. Owing to the variability of total cAMP produced among the assays, background counts were subtracted from raw values and activation data (with s.e.m.) were normalized to maximal activity within each experiment. *P*<0.0001 by ANOVA. (B) Differentiated WT-CAR1 (*n* = 2) and CM1234 (*n* = 2) cells were stimulated at 22°C with 5 µM 2′deoxy-cAMP in the presence of 10 mM DTT. Total cAMP (with s.e.m.) was measured at the times indicated. A plateau in cAMP accumulation for WT-CAR1 cells corresponds to ACA adaptation observed at ∼1 minute (see Fig. 4A). *P*<0.0001 by ANOVA. C. Differentiated WT-CAR1 and CM1234 were stimulated at 22°C with varying concentrations of cAMP, as indicated. To accurately measure dose responses, a single, differentiated cell population was stimulated with the indicated concentration of cAMP, with a 20 second time lag between separate assays (see the Materials and Methods). Experiments were repeated at least twice with duplicates for each time point. Values indicate means ± s.d. (D) WT-CAR1 and CM1234 cells were differentiated, and stimulated at 4°C with 100 nM cAMP in the absence or presence of 10 mM DTT, with a 20 second time lag between each assay. At the times indicated, lysates were prepared and ACA activity assayed for 1 minute at 22°C. (E) WT-CAR1 and CM1234 cells were differentiated and stimulated at 4°C with different concentrations of cAMP in the presence of 10 mM DTT, with a 20 second time lag between each stimulation. At the times indicated, lysates were prepared and ACA activity assayed for 1 minute at 22°C. (F) WT-CAR1 (circles) or CM1234 (boxes) cells were differentiated and stimulated at 4°C in the presence of DTT with the indicated concentrations of cAMP. After 1 minute, cells were lysed and assayed for ACA activity for 1 minute. Data from two experiments (open/closed symbols) are plotted with an averaged curve fit between the points.

If cAMP production was altered in CM1234 cells, several potential regulatory paths might be affected: basal ACA activity might be elevated, cAMP response could be hyperactive or adaptive responses attenuated. To assess these parameters, we directly measured ACA activity in suspension culture after response to a saturating cAMP stimulus. In WT-CAR1 cells, ACA was rapidly activated, with enzymatic activity peaking ∼1 minute after stimulation, followed by a return to basal levels by ∼6 minutes (Fig. 4A). Upon stimulation of cells with a CAR1 agonist, WT-CAR1 cells showed near linear accumulation of cAMP for ∼1 minute, before a plateau was reached, consistent with the adaptation of ACA activity (Fig. 4B). In CM1234 cells, ACA activity rapidly rose to levels similar to that in WT-CAR1 cells, but in contrast, to WT-CAR1 cells, ACA activity in CM1234 cells remained elevated throughout the experimental time course (Fig. 4A). Not surprisingly, the non-adapted ACA activity in CM1234 cells led to the continuous accumulation of cAMP during the entire time course (Fig. 4B).

We next assessed ACA activity in response to various cAMP concentrations. ACA activity in WT-CAR1 cells exhibited a rapid and transient, dose-dependent response to exogenous cAMP stimulation (Fig. 4C). By contrast, although basal ACA activity in CM1234 cells was similar to WT-CAR1 cells at the start of the assay (see Fig. 4B), ACA activity did not respond in a dose-dependent fashion, but reached similar maxima regardless of the initial stimulation concentration (Fig. 4C). From these findings we speculate that, in the absence of ACA adaptation, cAMP-stimulated CM1234 cells can be further activated in response to endogenously produced cAMP. We thus sought to measure ACA activity under conditions where endogenous cAMP production is minimized. At 4°C, CAR1-mediated pathways leading to ACA excitation and adaptation are partially responsive, with ACA activity per se delayed and reduced (Van Haastert, 1987b), therefore limiting the accumulation of endogenous cAMP. Accordingly, we stimulated WT-CAR1 and CM1234 cells at 4°C, but assayed ACA activity under standard conditions at 22°C. We also examined ACA response to continuous cAMP stimulation, by suppressing cAMP degradation using 10 mM DTT as a specific inhibitor of extracellular PDE (Orlow et al., 1981).

ACA response in WT-CAR1 cells at 4°C showed the standard pattern of transient activation, in the presence or absence of DTT (Fig. 4D). By contrast, when CM1234 cells were stimulated with cAMP at 4°C in the presence of DTT, to maintain a persistent non-varying signal, ACA activity did not adapt but remained continuously activated (Fig. 4D). When cAMP levels degraded in the absence of DTT, ACA activity in CM1234 cells returned to basal levels, indicating that elevated ACA activity in CM1234 cells results from defects in adaptation.

Finally, using DTT to suppress cAMP degradation, we assayed relative ACA activity in WT-CAR1 and CM1234 cells at 4°C in response to different concentrations of cAMP. ACA activity was not detected in the absence of cAMP in either cell line, but they exhibited nearly identical dose responses, with EC_50_ ∼7 nM cAMP (Fig. 4E,F) (Theibert et al., 1986). Thus, in the absence of CAR1 phosphorylation, basal and cAMP-stimulated ACA activities remain unchanged, but adaptive responses are defective.

**Fig. 5.**
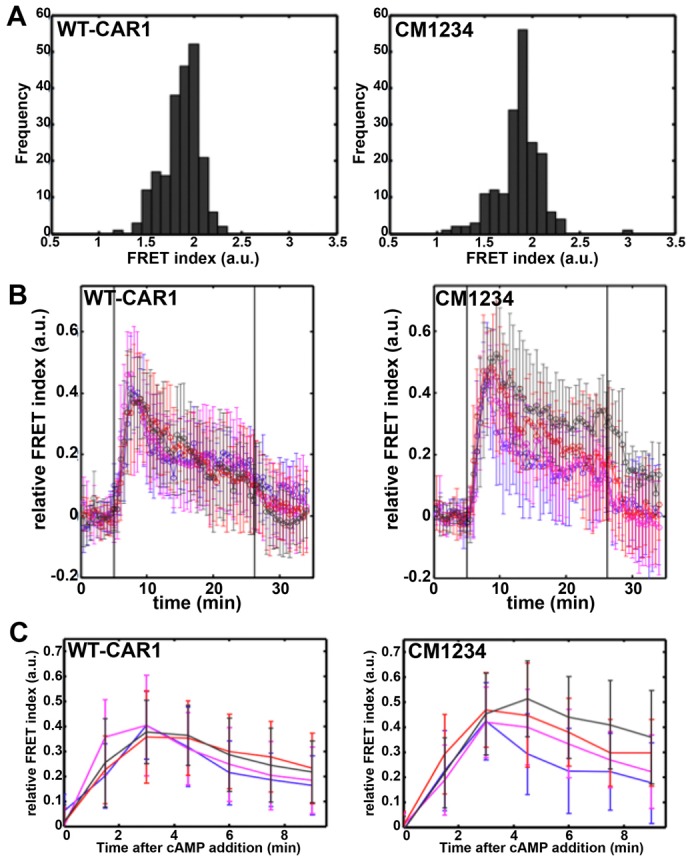
**Live-cell imaging of intracellular cAMP suggests defective adaptation of ACA in CM1234 cells.** (A) Histograms are shown for non-stimulated FRET ratios (a.u. = arbitrary units) as a function of numbers of WT-CAR1 and CM1234 cells (frequency) that express the cAMP sensor Epac1-camps. The time averages during the first 5 minutes prior to stimulation were obtained from each single-cell time series. The averages are 1.86±0.19 (s.d.) for WT-CAR1 cells and 1.86±0.23 (s.d.) for CM1234 cells and correspond to ∼400 nM basal cAMP for each cell type. (B) WT-CAR1 or CM1234 cells were stimulated with a constant cAMP concentration in a perfusion chamber using a pair of syringe pumps. The flow rate was kept constant at 2 ml/minute. Cells attached to a glass surface were first exposed to buffer without cAMP. Time-lapse recording (*t* = 0 minute) was started after 5–7 minutes. After an additional 5 minutes of plain buffer flow, the pump source was switched to buffer containing cAMP (black vertical line at *t* = 5 minutes) at 1 nM (blue), 10 nM (magenta), 100 nM (red) and 1 µM (black). The input flow was switched back to plain buffer after 25 minutes; the second black vertical line indicates the time (*t*∼27 minutes) when the concentration of exogenous cAMP had decreased by a factor of 1/*e*. The time-plots are relative FRET averages (a.u. = arbitrary units) from more than eight cells for each condition. Error bars indicate s.d. at each time point. (C) Data from Fig. 5B were time-binned at 1.5 minutes intervals. cAMP doses were at 1 nM (blue), 10 nM (magenta), 100 nM (red) and 1 µM (black). Error bars indicate s.d. at each time point.

To examine the altered response of CM1234 cells at a finer time-scale resolution, we quantified internal cAMP levels in cells expressing the FRET sensor Epac1-camps (Nikolaev et al., 2004; Gregor et al., 2010). Corresponding WT-CAR1 and CM1234 cells were plated in a perfusion chamber at a very low density, under continuous buffer flow in the presence or absence cAMP. These conditions allow the precise examination of individually isolated cells that have been effectively separated from any endogenously secreted cAMP or PDE. The basal level of cAMP in CM1234 cells before cAMP stimulation was, on average, quantitatively identical to that of WT-CAR1 cells (Fig. 5A), supporting our previous conclusions that basal ACA activity is unaltered in CM1234 cells.

Upon stimulation of WT-CAR1 cells with a non-varying cAMP concentration, we observe a transient increase in cytosolic cAMP, regardless of the stimulatory dose (Fig. 5B). The intracellular cAMP patterns have several phases. First, there is a rapid increase in intracellular cAMP, peaking at ∼2.5 minutes after the cAMP stimulus; this is followed by a rapid reduction in cAMP over a short period, with a more gradual decline in cAMP during the next 15 minutes. Upon removal of the exogenous cAMP stimulus, intracellular cAMP returned to basal levels. When the data are averaged and binned over 1.5 minute intervals during the first 9 minutes of stimulation (Fig. 5C), we observe cAMP patterns that are highly reflective of the biochemical assays (see Fig. 4). These data are consistent with a transient cAMP/CAR1-mediated activation of ACA that is rapidly adapted if the stimulatory dose of cAMP is maintained.

CM1234 cells respond to a 1 nM cAMP stimulation in a similar manner to WT-CAR1 cells (Fig. 5B). However, as the extracellular cAMP stimulus is increased, the FRET response peak becomes increasingly extended and broadened. Although no difference in the initial rate of cAMP increase is observed, the rate of cAMP attenuation in CM1234 cells is severely diminished, in a dose-dependent manner (Fig. 5C, see supplementary material Fig. S4 for statistical analysis). Furthermore, as the CM1234 cells are released from the cAMP stimulus (Fig. 5B, *t*>25 minutes), cytosolic cAMP levels begin to return to basal levels, indicating a suppression of ACA activity upon the removal of the exogenous cAMP stimulus. When the time-plots are binned at 1.5 minute intervals (Fig. 5C), they compare quite well with the CM1234 biochemical assays (Fig. 4). It should be emphasized that because intracellular cAMP is secreted continuously, the FRET signal would reflect more an approach to steady state than simple accumulation. Thus, the intra- and extracellular cAMP curves are not predicted to be quantitatively identical (see Fig. 4B).

In summary, basal cAMP levels and the rate of cAMP-stimulated synthesis of cAMP in CM1234 cells are unchanged compared with that of WT-CAR1 cells, but the timing and the magnitude of cAMP suppression are severely impaired in CM1234 cells. Collectively, these data are most consistent with the idea that CAR1 phosphorylation regulates ACA adaptation during persistent cAMP stimulation.

### Receptor phosphorylation does not regulate adaptation of the TORC2 and PI3K pathways, but promotes persistent ERK2 activity

The TORC2 and PI3K–CRAC pathways are transiently activated by cAMP in WT cells and are independently required for the CAR1-mediated activation of ACA (Insall et al., 1994a; Lee et al., 2005). We therefore analyzed the temporal dynamics of cAMP-mediated TORC2 and PI3K activation and adaptation in WT-CAR1 and CM1234 cells.

**Fig. 6.**
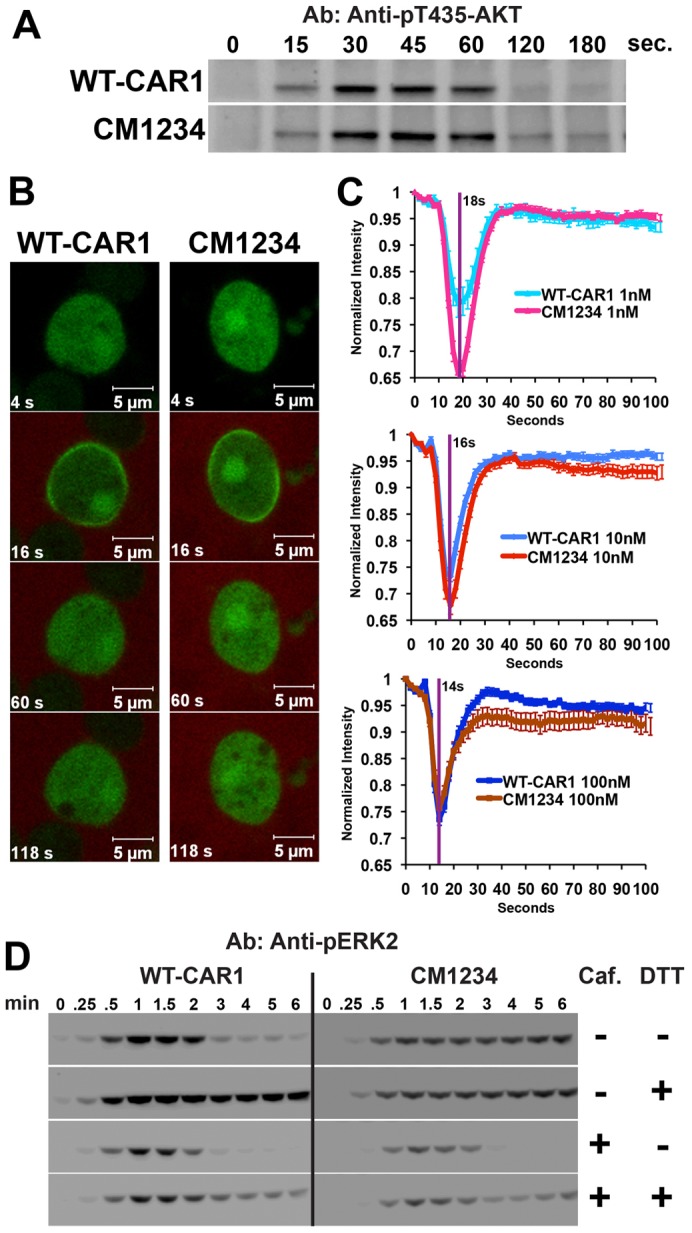
**Loss of CAR1 phosphorylation does not alter regulation of rapidly adapting pathways, but elevated cAMP production promotes persistent ERK2 phosphorylation.** (A) Differentiated WT-CAR1 or CM1234 cells were stimulated with 1 µM cAMP. Cells were lysed at the indicated times and pT435 AKT was assayed by immunoblot. (B) Differentiated WT-CAR1 or CM1234 cells expressing PH_CRAC_–GFP were exposed to a 10 nM global cAMP stimulus mixed with Alexa Fluor 633 dye to visualize the precise time of stimulation. Images are cells before [4 seconds (s)] and following the application of the 10 nM cAMP stimulus; the peak response is at 16 seconds. (C) PH_CRAC_–GFP response curves of differentiated WT-CAR1 or CM1234 cells to various cAMP concentrations, as indicated. Maximal response times are indicated with a vertical purple line in seconds (s). Values (with s.e.m.) are shown for three combined experiments. ANOVA was applied on all curves. The peak response time was the same between strains for each concentration and no difference in adaptation was observed. A difference in the magnitude of the response for the 1 nM (*P*<0.0001; WT-CAR1 *n* = 15, CM1234 *n* = 43) and 10 nM (*P*<0.0001; WT-CAR1 *n* = 30, CM1234 *n* = 31) stimulations was observed, but not for the 100 nM stimulation (*P* = 0.623; WT-CAR1 *n* = 26, CM1234 *n* = 29). (D) WT-CAR1 and CM1234 cells were differentiated and stimulated at 22°C with 10 nM cAMP to ensure the action of PDE was not masked by a saturating stimulation. Stimulations were performed in the absence or presence of 10 mM DTT, an inhibitor of secreted PDE, and in the presence or absence of 2.5 mM caffeine (Caf), an inhibitor of endogenous cAMP production. Conditions are indicated. At the times indicated, lysates were prepared and pERK2 assayed by immunoblot using anti-phospho-threonine/tyrosine ERK2 antibody. Experiments were repeated twice; representative blots are shown.

TORC2 kinase activity was monitored by assessing the phosphorylation of T435 in AKT, a TORC2 target site, following a uniform cAMP stimulation (Liao et al., 2010). We found that T435 of AKT is rapidly phosphorylated in response to cAMP in both WT-CAR1 and CM1234 cells, and then similarly dephosphorylated (Fig. 6A), suggesting that CAR1 phosphorylation does not regulate TORC2 adaptation. PI3K activity was monitored by visualizing the relocalization of PH_CRAC_–GFP following a global cAMP stimulus (Parent et al., 1998; Xu et al., 2006). We observed similar rapid, transient increases in PIP_3_/PH_CRAC_–GFP at the plasma membrane and concomitant depletion in the cytosol in both cell types (Fig. 6B). Although there might be a difference in the magnitude of the PI3K response at low cAMP concentrations, both cell lines exhibited similar dose-dependent temporal translocation changes (Xu et al., 2005) to and from the plasma membrane in response to cAMP (Fig. 6C), indicating that receptor phosphorylation is not required for adaptation of the rapid PI3K response. No differences between WT-CAR1 and CM1234 cells were observed in their ability to localize PIP_3_/PH_CRAC_–GFP in the direction of an applied cAMP gradient in individual, immobilized cells (supplementary material Fig. S5) or while migrating toward a point source of cAMP (supplementary material Movies 12 and 13).

We previously showed that phosphorylation of ERK2 does not adapt when WT cells are presented with a continuous extracellular cAMP stimulus; pERK2 levels only decline upon ligand depletion (Brzostowski and Kimmel, 2006). Because CM1234 cells exhibit enhanced cAMP signaling, we assessed the role of CAR1 phosphorylation on ERK2 regulation. ERK2 phosphorylation was monitored in WT-CAR1 and CM1234 cells stimulated with cAMP in the absence or presence of the PDE inhibitor DTT. ERK2 phosphorylation was transient in WT-CAR1 cells in the absence of DTT, but persistent in the presence of DTT, which maintains cAMP stimulation (Fig. 6D; supplementary material Fig. S6). By contrast, pERK2 levels in CM1234 cells persisted regardless of PDE inhibition (Fig. 6D; supplementary material Fig. S6), consistent with the continuous synthesis and accumulation of cAMP that we propose occurs in CM1234 cells. As expected, when endogenous cAMP synthesis is inhibited with caffeine in CM1234 cells and the cAMP input stimulus is allowed to degrade in the absence of DTT, pERK2 is transient (Fig. 6D; supplementary material Fig. S6). When the cAMP stimulus in caffeine-treated cells is maintained by inhibiting the extracellular PDE with DTT, persistent pERK2 is observed in both WT-CAR1 and CM1234 cells (Fig. 6D; supplementary material Fig. S6). Together, these data show that elevated cAMP production in CM1234 cells leads to persistent ERK2 activity.

### Receptor phosphorylation regulates F-actin assembly

Although loss of long-range signal relay and ACA adaptive response disrupts territory formation and aggregation in CM1234 cells, these defects do not directly explain the observed chemotactic abnormalities (see Fig. 1). Potentially, actin polymerization processes are altered in CM1234 cells. Unstimulated WT cells have basal F-actin levels at the plasma membrane and, within a few seconds after a global cAMP stimulus, F-actin is rapidly, but transiently, enriched along peripheral extensions of the plasma membrane. This primary F-actin response (at ∼12 seconds) is followed by a rapid depolymerization of F-actin from the cell periphery (at ∼15 seconds) and a secondary, transient F-actin polymerization response (at ∼20–25 seconds), which correlates with pseudopod extension and directional cell movement (Futrelle et al., 1982; Ma et al., 2004).

**Fig. 7.**
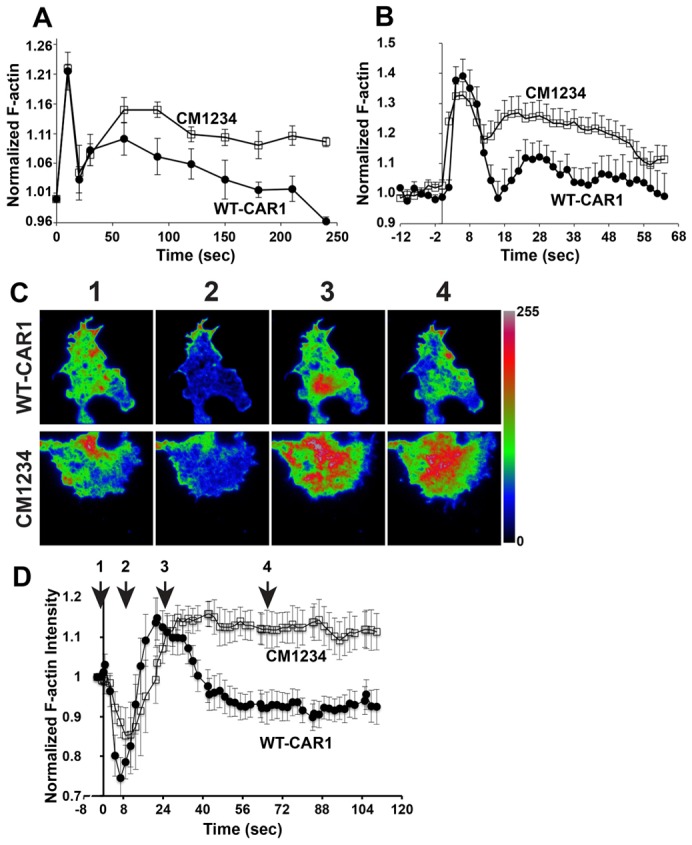
**Secondary F-actin responses to global cAMP attenuate in WT-CAR1 cells, but not in CM1234 cells.** (A) Differentiated WT-CAR1 (*n* = 4 experiments) and CM1234 (*n* = 5 experiments) cells received a global dose of 1 µM cAMP. MFI was measured by fluorometry and normalized to basal F-actin activity (0 seconds) in total cells fixed and stained with TRITC-phalloidin. ANOVA analysis indicates a significant difference between curves (*P* = 0.03 by ANOVA). (B) Differentiated WT-CAR1 and CM1234 cells expressing the ABD–GFP F-actin probe received a global dose of 1 µM cAMP (0 seconds). Optical sections of cells were imaged at 2 second intervals using a 100× objective lens by spinning-disk confocal microscopy. The Normalized F-actin response (see the Materials and Methods) and s.e.m. for WT-CAR1 (*n* = 18) and CM1234 cells (*n* = 20) from two separate experiments are plotted against time. ANOVA analysis indicates a significant difference between curves (*P* = 0.01 by ANOVA). (C) Differentiated WT-CAR1 and CM1234 cells expressing the ABD–GFP F-actin probe received a global dose of 1 µM cAMP mixed with the Alexa Fluor 633 dye (not shown), to track the application of the stimulus. Cells were imaged by TIR-FM at 2 second intervals using a 150× objective lens to capture F-actin polymerization dynamics proximal to the coverslip. Four representative events within the image sequence are delineated (see supplementary material Movies 15 and 16 to best view dynamics in gray scale): (1) Prestimulus condition; (2) Transient primary response showing loss of MFI in the contact area; (3) Secondary F-actin peak in the cell contact area; (4) Return to basal fluorescence intensity in WT-CAR1 cells and the continuation of F-actin activity in CM1234 cells. A rainbow LUT is used to highlight differences in fluorescence intensity on an eight-bit scale (scale shown). (D) Normalized average intensity change and s.e.m. in the entire contact area for WT-CAR1 (*n* = 11) and CM1234 cells (*n* = 10) from two separate experiments. The numbers above the graph approximately match the still images shown in Fig. 7C. ANOVA analysis indicates a significant difference between curves (*P*<0.0001 by ANOVA).

Global F-actin dynamics are easily measured in total cell extracts by phalloidin staining. Although both WT-CAR1 and CM1234 cells have a similar, rapid primary actin response after global stimulation with 1 µM cAMP (Fig. 7A), the secondary actin rise in CM1234 cells trends to a more extensive and more persistent response compared with that observed in WT-CAR1 cells. These data extend previous observations in CM1234 cells (Kim et al., 1997) and are consistent with the extensive turning behavior observed in our chemotaxis assays (see Fig. 1; supplementary material Figs S2, S3).

Because phalloidin assays are unable to identify subcellular compartmentalization, we examined cAMP-mediated F-actin polymerization in live WT-CAR1 and CM12324 cells expressing the F-actin probe ABD–GFP (Pang et al., 1998; Bretschneider et al., 2004; Khurana et al., 2005; Yan et al., 2012) using 4D spinning-disk confocal microscopy and total internal reflection-fluorescence microscopy (TIR-FM); the latter is an imaging modality that can quantify F-actin changes at the area of cell contact with the coverslip (Yan et al., 2012).

Using spinning-disk confocal microscopy, we captured rapid optical slices in the *Z* plane (∼15 *Z*-slices in 750 mseconds), before and after the application of a global 1 µM cAMP stimulus. Optical slices were combined to create a maximum intensity projection of F-actin activity over time, as measured by ABD–GFP fluorescence. Both WT-CAR1 and CM1234 cells exhibited a rapid, but transient, primary F-actin response. However, similar to the phalloidin measurements, the secondary F-actin response of CM1234 cells was enhanced relative to that of WT-CAR1 cells (Fig. 7B and supplementary material Movie 14; compare with Fig. 7A).

In TIR mode, the primary F-actin response of *Dictyostelium* to cAMP is observed as a depletion of ABD–GFP mean fluorescence intensity (MFI) at the cell contact area, which is followed by a transient increase in MFI, marking the secondary F-actin response (see Fig. 7C,D) (Yan et al., 2012). When cells were stimulated globally with 1 µM cAMP and the incorporation of the ABD–GFP probe into dynamic actin structures was measured by TIR-FM, both WT-CAR1 and CM1234 cells followed the same primary response with a loss of MFI in the contact area (see point 2 in Fig. 7C,D; supplementary material Movie 15). However, in stark contrast to WT-CAR1 cells, the kinetics of the secondary F-actin response in CM1234 cells, as with the experiments described above (see Fig. 7A,B), was greatly prolonged (Fig. 7C,D; supplementary material Movie 16).

Together, these data argue that CAR1 phosphorylation is required to dampen the secondary actin response, which, when lacking, leads to the loss of lateral pseudopod suppression and promotes excessive turning behavior during chemotaxis of CM1234 cells (Soll et al., 2009). In this context, although CM1234 cells are able to polymerize actin for pseudopod extension (see Fig. 1; supplementary material Movies 4 and 5), they do not polarize toward cAMP to the same extent as WT-CAR1 cells do, which gives rise to chemotactic defects (see Fig. 1).

## Discussion

We have demonstrated that the absence of CAR1 phosphorylation has significant regulatory effects on chemotaxis and cAMP signal relay. Phosphorylation of CAR1 in *Dictyostelium* is required for persistent directional movement, attenuation of F-actin polymerization, and cAMP wave production. The cAMP-dependent pathways, PI3K, TORC2 and the primary actin responses, which activate and deactivate rapidly (∼30 seconds), precede CAR1 phosphorylation and appear unaffected by the phosphorylation state of CAR1. Conversely, the delayed ACA and ERK2 activities and secondary actin polymerization, which occur concomitantly with CAR1 phosphorylation, exhibit extended responses in cells expressing the non-phosphorylatable CM1234 CAR1 receptor.

In mammalian cells, GPCR kinases (GRK) regulate multiple receptor pathways (Pitcher et al., 1998; Ferguson, 2001; Evron et al., 2012). Arrestin proteins bind to phosphorylated cytoplasmic residues of GPCRs, which ablates signaling to heterotrimeric G proteins and promotes endocytic internalization (Shenoy and Lefkowitz, 2011; Shukla et al., 2011). Internalized receptors can be recycled to the plasma membrane or targeted to lysosomes for downregulation. However, more recent studies in mammalian cells suggest a direct role for arrestin scaffolding of ERK, cofilin and PI3K to chemotactic GPCRs in the regulation of actin assembly and chemotaxis (Kraft et al., 2001; Fong et al., 2002; Su et al., 2005; Sai et al., 2006; Ueda et al., 2006; Vroon et al., 2006; Cotton and Claing, 2009; Zidar et al., 2009; Décaillot et al., 2011; Min and Defea, 2011). More remarkably, the actin defects in CM1234 cells correlate well with those of neutrophils that have diminished BTL1 receptor phosphorylation (Gaudreau et al., 2002; Kavelaars et al., 2003; Vroon et al., 2004). GPCR kinase 6 (GRK6) is the primary kinase for the BTL1 receptor and, similar to CM1234 cells, GRK6^−/−^ neutrophils display prolonged actin polymerization and abnormal chemotactic progression in response to ligand stimulation.

A simple mechanism that links GPCR internalization with positive or negative regulation of chemotaxis in *Dictyostelium* has not emerged. Exposure to a saturating dose of cAMP will downregulate CAR1 from the cell surface (Van Haastert, 1987a) and recent evidence, using single-particle imaging techniques, suggests that a fraction of cell surface CAR1 is internalized by a process that requires receptor phosphorylation and occurs within minutes of a global stimulus (Sergé et al., 2011). Although a group of proteins with arrestin-like domains has been identified in *Dictyostelium* (Guetta et al., 2010), none have been studied for associative roles with CAR1 or analyzed in the context of chemotaxis. In addition, whereas ∼80% of high-affinity WT CAR1 sites are lost upon cAMP stimulation, most high-affinity CAR1 sites are maintained in CM1234 cells (Caterina et al., 1995). If the loss of high-affinity CAR1 sites were required to promote ACA adaptation, the persistence of high-affinity cAMP binding in CM1234 cells might antagonize the process.

The conclusions regarding a role for CAR1 phosphorylation in the regulation of actin assembly and ACA adaptation can be placed in a broader signaling context that associates PKA function and chemotaxis. *Dictyostelium* with elevated cAMP (Wessels et al., 2000) or constitutively active PKA (Zhang et al., 2003) are effective physiological mimics of CM1234 cells. Accordingly, hyperactive PKA cells have similar chemotaxis defects to those seen in CM1234 cells, with enhanced lateral pseudopod formation and reduced ability to propagate long-range cAMP signaling (Sawai et al., 2005). The persistent activation of ERK2, a presumed inhibitor of cAMP degradation (Maeda et al., 2004), in CM1234 cells would further serve to accentuate PKA hyperactivity and reinforce the connections among these various signaling mutants and their shared loss of lateral pseudopod suppression.

We suggest a feedback-dampening mechanism that interconnects receptors, actin, ACA, PKA and intra- and extracellular cAMP levels, that functions independently of pathways immediately upstream of ACA. Indeed, these multiple adaptive/de-adaptive pathways are in approximate temporal concordance with the phosphorylation (and dephosphorylation) kinetics of CAR1. Conversely, the rapidly activating/deactivating paths, such as PI3K, TORC2 and AKT/PKBR1, which are required for ACA activation, respond before CAR1 phosphorylation and are notably unaltered by the absence of CAR1 phosphorylation in CM1234 cells. In addition, phosphorylated CAR1 might still promote Gα2/βγ dissociation (Janetopoulos et al., 2001), possibly indicating that many G-protein signaling pathways immediately downstream of CAR1 are also not influenced by phosphorylation.

Previously, we suggested that pathways involving Gα9 (Brzostowski et al., 2004) and PI3K (Comer and Parent, 2006) influence adaptation of ACA. In particular, whereas minimal PI3K activity might direct full ACA activation, WT PI3K levels appear to be required for ACA adaptation. These multiple signaling paths could act separately or converge at a common regulatory target. Regardless, the individual loss of Gα9, PI3K or pCAR1 would disrupt ACA adaptation, even if the other paths were fully active. Our data thus underscore the complexity of adaptive signaling in *Dictyostelium* and are not simply consistent with models that suggest a possibility for a single global pathway (Takeda et al., 2012). Crossregulation by receptor phosphorylation and possibly involving arrestin-related pathways and multiple kinases supports a commonality of chemotactic regulation in *Dictyostelium* and the metazoa.

## Materials and Methods

### Cell culture and development

*car1*^−^/*car3*^−^ cells (strain RI9) and G418-selectable extrachromosomal expression vectors using pJK1 for WT-CAR1 and the CAR1-CM1234 variant (Caterina et al., 1994; Hereld et al., 1994; Kim et al., 1997) were kindly provided by Dr Peter Devreotes (Johns Hopkins University School of Medicine) and the RI9 strain was also provided by the Dicty Stock Center. Expression vectors were electroporated into RI9 cells and populations of the designated stains, WT-CAR1 and CM1234, were differentiated on agarose. Sori were picked and spores were pooled to select for cells that completed the developmental cycle for use in subsequent experiments. Receptor expression levels were verified by immunoblot assay. The WT-CAR1 and CM1234 were derived multiple times during the course of experimentation and phenotypes were confirmed to be the same.

WT-CAR1 and CM1234 cells were transformed with G418-selectable extrachromosomal expression vectors for PH_CRAC_–GFP or ABD–GFP (ABD–GFP kindly provided by Dr Annette Muller-Taubenberger, Ludwig Maximilians University, Munich). When necessary, fluorescent cells were sorted by flow cytometry.

RI9 cells expressing WT-CAR1 or CM1234 receptors were grown at 22°C in D3-T medium (KD Medical, Columbia, MD) by shaking at 200 rpm in Erlenmeyer flasks to log phase (1–3×10^6^ cells/ml), in the presence of 50 µg/ml G418. For experiments requiring nutrient deprivation to promote differentiation, growing cells were harvested and washed twice with Development Buffer (DB; 7.4 mM NaH_2_PO4-H_2_O, 4 mM Na_2_HPO_4_-7H_2_O, 2 mM MgCl_2_, 0.2 mM CaCl_2_, pH 6.5). For biochemical assays, washed cells were suspended in DB at 2×10^7^ cells/ml, shaken at 100 rpm for 1 hour and then supplemented to 75 nM cAMP every 6 minutes for 4–5 hours, to promote synchronous differentiation. To observe dark-field waves or developmental structures (see Imaging), washed cells were suspended in DB and plated at a final density of 6×10^5^ cells/cm^2^ on a 1.4% agarose surface with DB in 6- or 12-well plates. Cells were permitted to attach for 15 minutes before removing the overlaying DB by aspiration. Residual DB was evaporated for 2 minutes and the plate lid was replaced and cells were incubated at 22°C. Submerged development assays were carried out in 24-well plates. Washed cells suspended in 300 µl of DB were plated at a final density of 1.25×10^5^ cells/cm^2^ and were allowed to adhere for 15–30 minutes before wells were imaged by time-lapse microscopy at 22°C (see Imaging).

### Cell and developmental imaging

#### Stereoscope

Developmental structures formed on agarose surfaces were imaged using a Leica MZ125 stereoscope and a 0.5× plan objective at 8× magnification with a Roper CoolSnap HQ CCD camera.

#### Submerged development assay

Under buffer (DB) development in multiwell plates was imaged by bright-field using an Olympus IX81 inverted microscope and a 10×, 0.3 NA Uplan Fl objective lens, with a Photometrics (Tucson, AZ) 1024 Cascade IIB EMCDD in standard gain mode. Time-lapse images of differentiation were captured every 5 minutes for 12–18 hours. The automated stage (Applied Scientific Instrumentation, Eugene, OR) and focus were controlled with MetaMorph software (Molecular Devices, Sunnyvale, CA) over the time lapse.

#### EZ-TaxiScan

Chemotaxis assays were carried out using the EZ-TAXIScan chamber (Effector Cell Institute, Tokyo, Japan) assembled according to the manufacturer's procedures on a non-coated glass surface. Up to six conditions could be assayed in one multi-well chamber assembly. A chamber consists of two wells situated opposite one another across a terrace 260 μm in length under which cells crawl from the starting well to an opposing well containing chemoattractant. Approximately, 2000 cells were loaded into the starting well and buffer was drawn from the opposing well to cause flow. Once more than 100 cells accumulated along the starting border of the terrace, flow was stopped by reintroducing buffer into the opposing well. cAMP was added to the opposing well at various concentrations. A stable linear gradient forms by diffusion within 10 minutes and is maintained for ∼1 hour before gradually decreasing. After the addition of chemoattractant, image sequences were captured. Assays were performed simultaneously for differentiated WT-CAR1 and CM1234 cells against the indicated chemoattractant and caffeine concentrations. Migration was recorded with 15 second intervals for 30–45 minutes at 22°C.

#### Development on agarose surfaces

Development on agarose was imaged using an Olympus IX81 inverted microscope and a phase 2 condenser ring paired with a non-phase-contrast 2×, 0.08 NA PlanApo N objective lens, to achieve a pseudo-dark-field effect. Images for individual wells were captured every 30 seconds for 12–18 hours with a Photometrics 1024 Cascade IIB EMCDD in standard gain mode binned twice to observe dark-field cell shape changes. The stage position and focus were controlled automatically with MetaMorph software. Up to four conditions (wells) were imaged for a single experiment due to the limited speed of the stage motor to visit each well within the 30 second interval. Imaging and wavelet analyses of dark-field oscillations on agarose surfaces were as described (Sawai et al., 2005; Sawai et al., 2007).

#### PH_CRAC_–GFP dynamics

Differentiated cells were plated in eight-well Lab-Tek II coverglass-bottomed chambers (Nalge Nunc, Rochester, NY) at ∼50×10^3^ cells/cm^2^ in DB containing 2.5 mM caffeine in a volume of 300 µl. Alexa Fluor 633 dye (Invitrogen, Grand Island, NY), 20 µg/ml final concentration, was mixed with cAMP to monitor the application of the stimulus. Cells were pretreated with 1 µM Latrunculin B (Invitrogen) for 10 minutes prior to stimulation to minimize cell movement for intensity analysis. A 100 µl volume of cAMP and Alexa Fluor 633 mixture was applied to each well during a time series acquisition to achieve the final cAMP concentration indicated. The resultant transient cytosolic to membrane translocation of PH_CRAC_–GFP was visualized using an inverted Zeiss 710 confocal microscope and a 40×, 1.3 NA ECPlan-NEOFLUAR objective lens, with a 2 second time-lapse interval for ∼3 minutes. GFP and Alexa Fluor 633 were excited with a 488 nm and 633 nm laser and detected using a 500–550 nm and 640–700 nm spectral range, respectively.

For chemotaxis assays, a micropipette containing 1 µM cAMP mixed with Alexa Fluor 633 dye was used. Cells were imaged at ∼1.5 second intervals with an inverted Zeiss 780 confocal microscope with the same optics and filters noted above. In some cases, the needle was moved to observe changes in the direction of the PIP_3_ marker.

#### Actin dynamics in live cells using 4D spinning-disk confocal microscopy

Differentiated cells were plated in eight-well Lab-Tek II coverglass-bottomed chambers at ∼50×10^3^ cells/cm^2^ in DB containing 2 mM caffeine in a volume of 300 µl. A 100 µl volume of cAMP was applied to each well during a time series acquisition to achieve a final 1 µM cAMP concentration. Images were captured using an Olympus IX81 inverted microscope equipped with a 100×, 1.45 NA UApo objective lens, and 512 Cascade IIB EMCDD in EM gain mode (Photometrics, Tuscon, AZ). ABD–GFP was excited with 488 nm light and the emitted light was collected through a 525/50 filter (Chroma, Bellows Falls, VT). At each 2 second time point, ∼15 optical sections at 500 nm were collected in the *Z*-axis, ranging from the bottom to top of the cell. Sections were collected in 50 msecond intervals using a piezo-electric stage (Applied Scientific Instrumentation, Eugene, Oregon).

#### TIR-FM

Differentiated cells were plated in eight-well Lab-Tek II coverglass-bottomed chambers at ∼50×10^3^ cells/cm^2^ in DB containing 2 mM caffeine in a volume of 300 µl. A 100 µl volume of cAMP and Alexa Fluor 633 mixture was applied to each well during a time series acquisition to achieve a final 1 µM cAMP concentration. Images were captured in TIRF mode using an Olympus IX81 inverted microscope and a 150×, 1.45 NA UApo objective lens, with an additional 1.6× optovar magnification and 1024 Cascade IIB EMCDD in EM gain mode. Fluorophores were simultaneously excited with 488 and 640 nm light and the emitted light was passed through an optical splitter (DualView, Photometrics, Tuscon, AZ) with a 565 nm beam splitter. The split signal was passed respectively through a 525/50 and 650 LP filter (Chroma, Bellows Falls, VT) to visualize the GFP and Alexa Fluor 633 signal on the right and left half of the EMCDD chip. Use of an autofocus photodiode accounts for the periodic (∼10 second) gaps that disrupt the 2 second interval in the time series.

#### cAMP dynamics

Changes in the level of cytosolic cAMP was measured in live WT-CAR1 or CM1234 cells expressing Epac1-camps (Nikolaev et al., 2004; Gregor et al., 2010). *Dictyostelium* were co-transfected with vectors that express either CAR1 (WT or CM1234) or Epac1-camps, and selected for growth in G418 (for CAR1) and hygromycin (for Epac1-camps). Axenically grown cells were washed twice and shaken for 4–6 hours at the density of 1.6×10^7^ cells/ml before plating in a perfusion chamber (Gregor et al., 2010). Cells settled for about 15–30 minutes before the buffer (DB) flow was initiated. A syringe pump (NE-1000X dual, New Era Pump Systems, NY) was used to create the flow at the rate of 2 ml/minute. Rates of buffer and stimulus flow were calculated in separate experiments using fluorescein as a dye (Gregor et al., 2010). Cells were washed for 7–10 minutes prior to image acquisition. Time-lapse recording was performed with an inverted epifluorescence microscope (IX81, Olympus), equipped with an oil immersion lens (60× UPlanApo NA 1.35, Olympus) as described (Gregor et al., 2010). Images were acquired every 30 seconds by an EM-gain CCD camera (Cascade II, Photometrics) using Metamorph software (Molecular Devices). Snapshots from three arbitrary regions in the same chamber each containing a single cell were imaged in serial using an automated stage. Typically, time delay between the images acquired from each position was within 5 seconds.

### Image analyses

#### Chemotaxis

Stacked TIFF images obtained from the EZ-TAXIScan system were converted to QuickTime movies in ImageJ (http://rsbweb.nih.gov/ij/) and cell shape changes were traced by a combination of automated and manual tracing and analyzed in the Dynamic Image Analysis System (DIAS; Solltech, Oakdale, IA) software package.

#### PH_CRAC_–GFP dynamics

The transient translocation of the PH_CRAC_–GFP probe after stimulation was measured using the Zeiss ZEN software package. A region of interest (ROI) was drawn on the interior of cells to measure the mean intensity change in the cytosol over time. Background intensity was measured in each frame and was subtracted from the mean ROI measurements from that frame. The appearance of the Alexa Fluor 633 dye in the red channel was used to align the time series to the stimulation. For each cell, the subtracted intensity measurements were normalized to the ROI measurement from the first frame. The normalized values were averaged for all cells.

#### cAMP dynamics

At time *t* and spatial coordinate ***r***, we define fluorescence intensity *I*_ex,em_ (***r***, *t*) where ‘ex’ and ‘em’ denotes excitation and emission wavelength respectively. Three channels *I*_410, 480_, *I*_410, 540_ and *I*_495, 540_ were acquired in serial at each time point. Exposure time was set between 15 to 30 mseconds. Camera EM-gain was fixed at 3500 for *I*_410, 480_ and *I*_410, 540_. For *I*_495, 540_, the gain was set to zero to avoid pixel saturation. A masked region 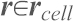 occupied by a single cell was defined at every time point by setting the threshold to the mean of the averaged masked and averaged unmasked regions. Next, we subtracted the background by calculating the signal intensity from the regions of interest as follows.
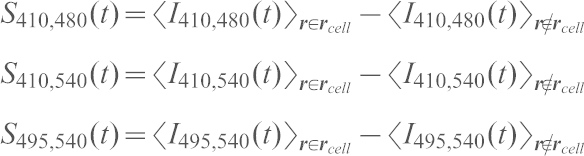
where < > denotes spatial averaging. To obtain the FRET index, the CFP/YFP channel fluorescence ratio was corrected for the spectral bleed-through (Börner et al., 2011). Namely,
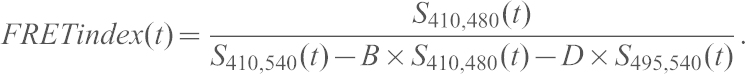
The fraction of bleed-through *B* ( = 0.54) and *D* ( = 0.60) were determined from independent measurements of WT cells expressing only CFP or YFP. Finally, we obtained the ‘Relative FRET index’ by subtracting the time average of the first 10 time points (5 minutes) from the FRET index. Cells that did not respond within 5 minutes of stimulus injection were removed from the dataset. Data were averaged over *n*≥8 cells (12 cells on average) for each condition and the relative changes in the FRET index from its pre-stimulus value were obtained and plotted as the average relative change in the FRET index.

#### Actin dynamics in live cells using 4D spinning-disk confocal microscopy

Optical sections were combined to create a maximum intensity projection image. Loss of fluorescence intensity as a result of acquisition photobleaching was corrected using the ImageJ Bleach Correction algorithm (http://fiji.sc/Bleach_Correction). Images were thresholded for intensity to the peak of primary actin response and the ‘Raw Area’ of F-actin polymerization was measured for the cell for each time interval. To compare between cells of different sizes, the Raw Area was normalized by the total cell area to yield the ‘Normalized F-actin Area’. The Normalized F-actin Area of the first ∼five unstimulated time points was averaged to yield the ‘Prestimulus F-actin Area’. To calculate the ‘Normalized F-actin Response’ over the time course, the ‘Normalized F-actin Area’ was divided by the ‘Prestimulus F-actin Area’ for each time point; these results were plotted against time.

#### TIR-FM

Changes in ABD–GFP fluorescence were measured with the MetaMorph software package. The area of the cell in contact with the coverslip was thresholded by intensity to exclude background fluorescence. The mean intensity was measured in the thresholded region over time with background intensities subtracted. For each cell, the subtracted intensity measurements were normalized to the measurement from the first frame. The normalized values were averaged for all cells.

### Biochemistry

#### Adenylyl cyclase assays

Adenylyl cyclase assays were modified from Theibert and Devreotes (Theibert and Devreotes, 1986). Differentiated cells were resuspended at 1×10^7^ cells/ml and shaken at 200 rpm in phosphate buffer (PB) containing 2 mM caffeine for 30 minutes to inhibit cAMP production for basal measurements. Cells were washed free of caffeine in cold phosphate buffer containing 2 mM MgSO_4_ and resuspended at 8×10^7^ cells/ml and maintained at 4°C prior to stimulation. For assays where direct comparisons between conditions (e.g. −/+ 10 mM DTT) were made, a given batch of washed cells was split and stimulated sequentially with a 20 second lag to allow for sample handling. Such assays required two experimenters to manipulate samples. Assays were performed entirely at 22°C or cells were stimulated at 4°C and lysates were measured for ACA activity at 22°C. For stimulation at 22°C, a 2 ml aliquot of cells was shaken for ∼1 minute to reach room temperature prior to stimulation. For stimulation at 4°C, cells were maintained and stimulated at 4°C; at specific points after stimulation, cells were lysed through a 5 µm Millipore filter into tubes containing [α-^32^P]ATP and incubated for 1 minute at 22°C. Accumulated [^32^P]cAMP was measured as described (Theibert and Devreotes, 1986).

For cAMP accumulation assays, differentiated cells were treated with caffeine, as above, washed, suspended at 5×10^7^ cells/ml in cold PB and maintained on ice prior to stimulation. A 2 ml aliquot of cells was shaken and allowed to reach 22°C (∼1 minute) and stimulated with 5 µM of the cAMP analog 2′deoxy-cAMP in the presence of 10 mM DTT. Aliquots of stimulated cells at specific time points were lysed in 3.5% perchloric acid and total cAMP was measured by competition assay (Amersham, TRK 432). 2′deoxy-cAMP does not compete with cAMP in the binding assay,

#### ERK2 and TORC2 phosphorylation assays

The phosphorylation state of ERK2 and AKT after chemoattractant stimulation in differentiated cells was assessed, respectively, by immunoblotting with an anti-phospho-threonine/tyrosine ERK2 antibody (Cell Signaling Technology, Beverly, MA; 1∶1000 dilution) and anti-phospho-PDK2/HM site (Cell Signaling Technology, Beverly, MA, 1∶1000 dilution), as previously described (Brzostowski and Kimmel, 2006; Liao et al., 2010).

#### CAR1 immunoblots

Proteins were separated and transferred to membrane using the Novex (Invitrogen) protein blotting system and probed with rabbit anti-CAR1 polyclonal antibody at a 1∶2000 dilution (gift from Peter Devreotes, Johns Hopkins University School of Medicine). Note that protein samples for CAR1 blots were not heated to avoid aggregation in sample wells.

#### F-actin assays

Differentiated cells were diluted to 1×10^7^ cells/ml and shaken at 200 rpm in phosphate buffer containing 2 mM caffeine for 30 minutes, and maintained in caffeine before stimulation. Cells were stimulated with 1 uM cAMP and were fixed and stained with TRITC-phalloidin and the level of F-actin was measured as a readout of MFI in a fluorometer. Stimulated samples were normalized against unstimulated sample over the time course.

### Statistical analyses

Student's *t*-test and ANOVA analyses were performed using the Prism 6 software package (Prism Software, Orange Co., CA). For ANOVA, the P value for the interaction effect between cell type or condition was reported.

## Supplementary Material

Supplementary Material
