# Supplementary material for: Phosphorylation of chemoattractant receptors regulates chemotaxis, actin reorganization and signal relay
Source: J Cell Sci. 2013 Oct 15;126(20):4614–26. doi: 10.1242/jcs.122952 (PMC3795335; doi:10.1242/jcs.122952)
Supplement: Supplementary Material [file supp_126_20_4614__index.html]

Phosphorylation of chemoattractant receptors regulates chemotaxis, actin reorganization and signal relay — Supplementary Material 

# Phosphorylation of chemoattractant receptors regulates chemotaxis, actin reorganization and signal relay

## JCS122952 Supplementary Material

**Files in this Data Supplement:**

- **Supplementary Material PDF**
