# Supplementary material for: Phosphorylation of chemoattractant receptors regulates chemotaxis, actin reorganization and signal relay
Source: J Cell Sci. 2013 Oct 15;126(20):4614–26. doi: 10.1242/jcs.122952 (PMC3795335; doi:10.1242/jcs.122952)
Supplement: Supplementary Material [file supp_126.20.4614_JCS122952.pdf]

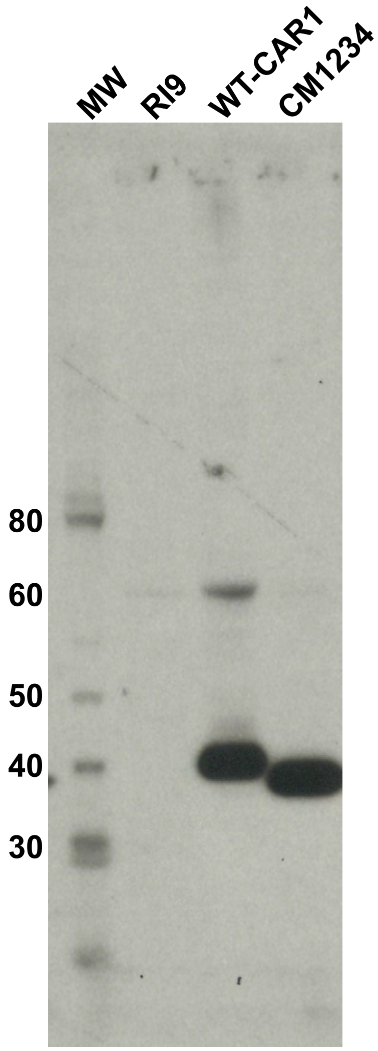

Fig. S1. WT-CAR1 and CM1234 protein are expressed at comparable levels. CAR1 variants were constitutively expressed in a parental strain (RI9) that lacks both CAR1 and CAR3 (Caterina et al., 1994). Equivalent loads of total protein lysates of the indicated strains were immunoblotted against α-CAR1.

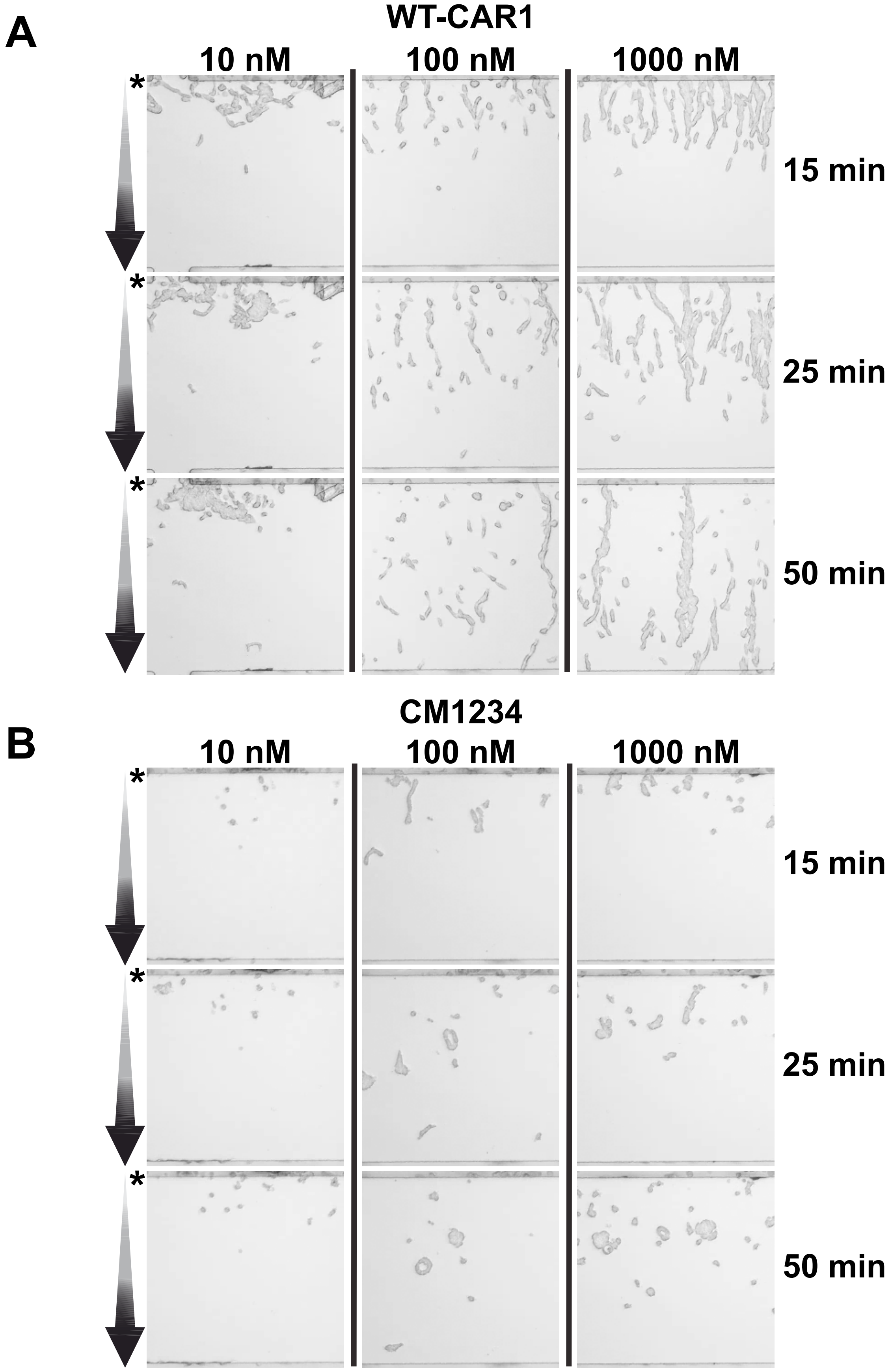

Fig. S2. Loss of CAR1 phosphorylation impairs directional migration within a linear cAMP gradient. Three frames (times indicated) from Movie 1 are shown for differentiated WT-CAR1 (A) or CM1234 (B) cells exposed to linear cAMP gradients established with the indicated concentration of cAMP in an EZ-TAXIScan chamber. The direction of increasing cAMP concentration is indicated with arrows. Origin of cells in chamber is indicated (*).

A

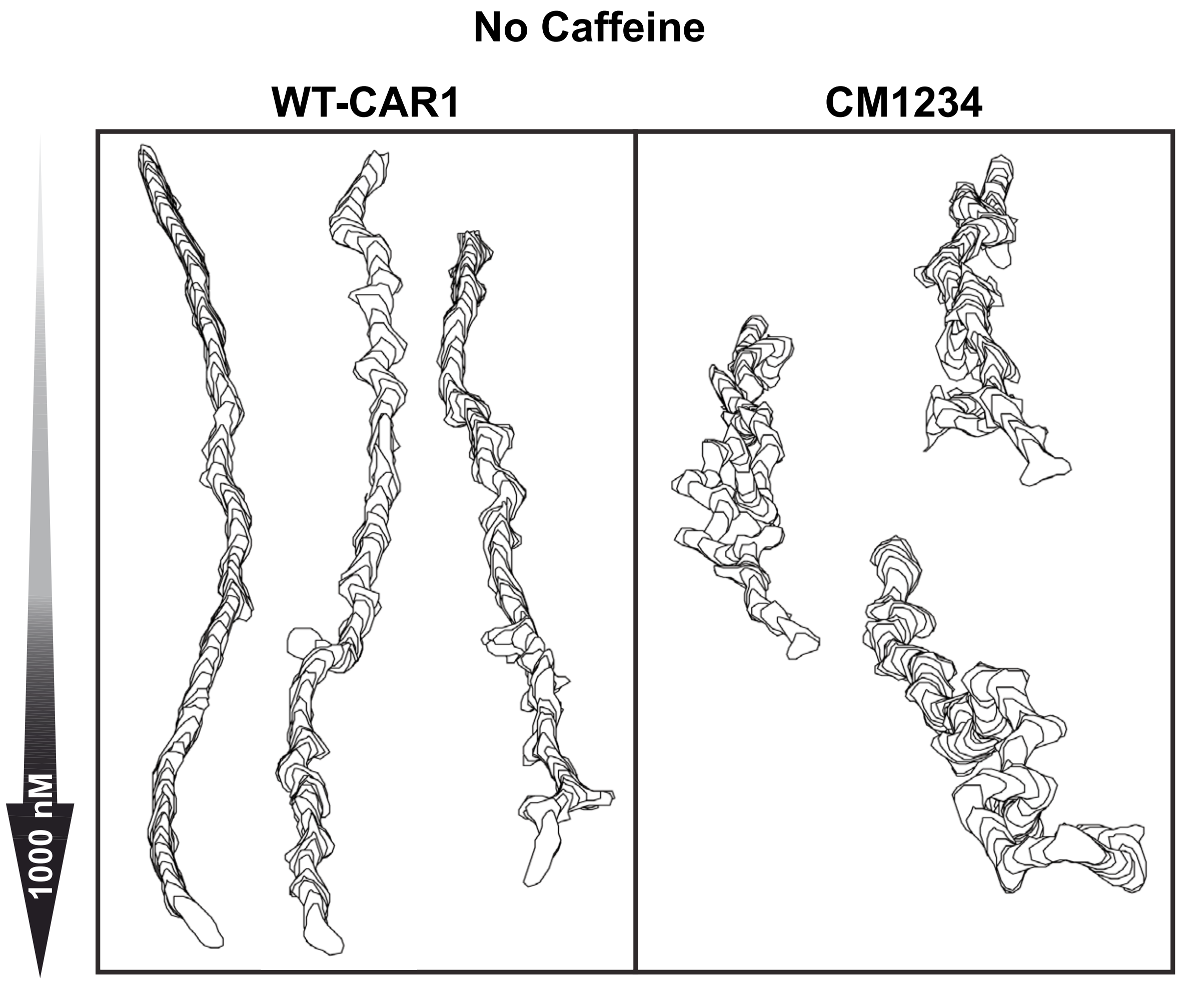

B

No Caffeine

1000 nM	WT-CAR1	CM1234	P value
Speed (um/min)	9.9 +/-0.56	9.0 +/-0.61	0.678
Directionality	0.89 +/-0.02	0.55 +/-0.07	<0.0001
Roundness (%)	68 +/-1.8	76 +/-1.53	0.003

Fig. S3. Loss of CAR1 phosphorylation impairs directional migration within a linear cAMP gradient in the absence of caffeine. A. While caffeine is used routinely in chemotaxis assays, assays were also performed in the absence of caffeine. Cells were seeded in EZ-TAXIScan chambers at low density and cell movement was analyzed to 1000 nM cAMP as in Figure 1. Similar to observations made in the presence of caffeine, CM1234 cells wandered more and were less polarized than WT-CAR1 cells; however, no enhancement of speed was observed. See Movies 6 & 7 for cell tracings. Shown are single cell outline traces using the DIAS software package. The cell tracings were stacked for 80 frames and compiled into one image to display morphometrics of the sequence. B. Metrics were determined from tracings of ~22 cells as described in Figure 1C. P values of student T tests are shown.

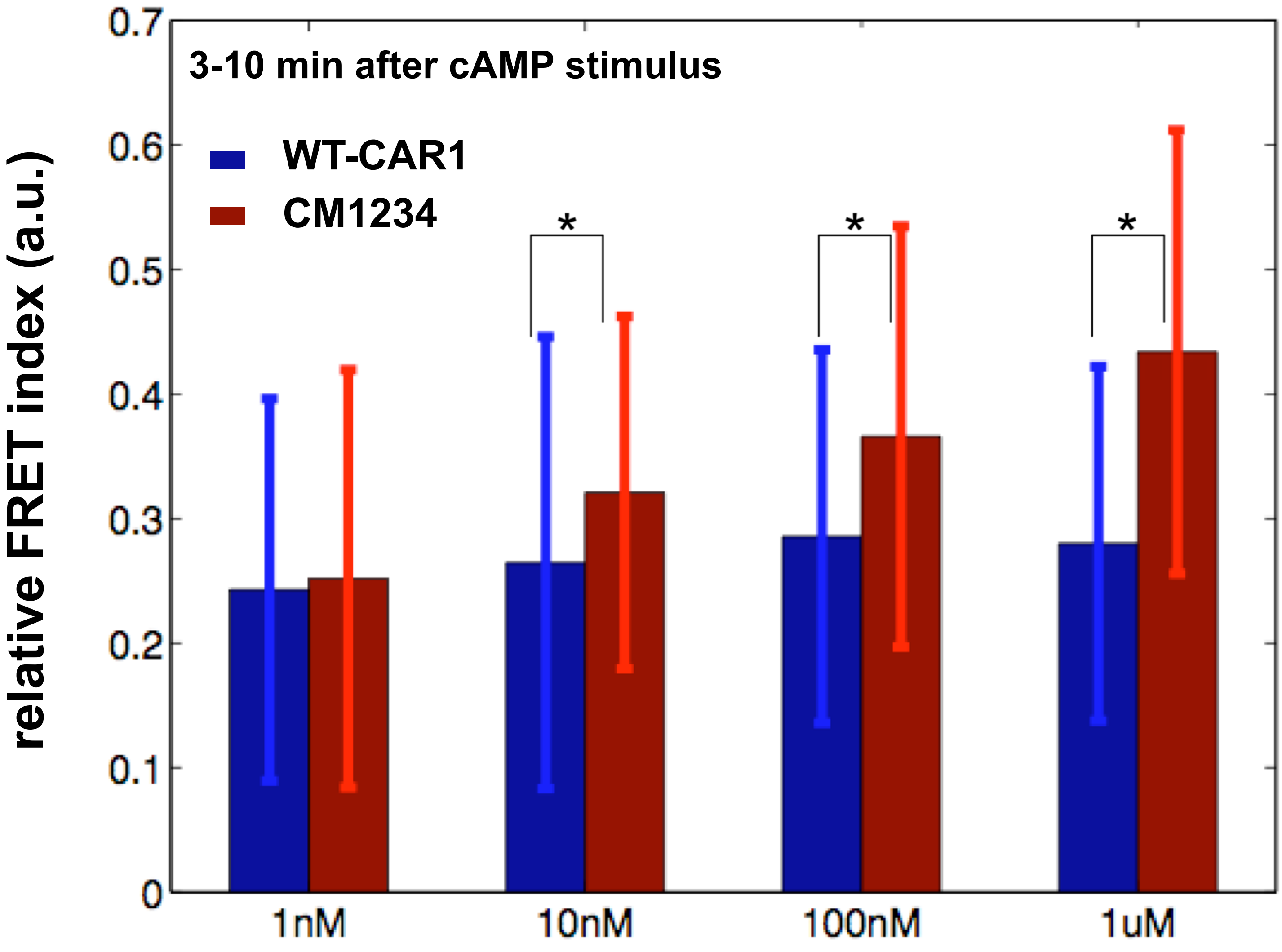

Fig. S4. Live cell imaging of intracellular cAMP suggests defective adaptation of ACA in CM1234 cells. A student t-test was applied to determine the statistical significance of the FRET response between WT-CAR1 and CM1234 cells during the indicated time window post stimulation for the various cAMP concentrations used in the experiment described for Figure 5B; *P<0.001.

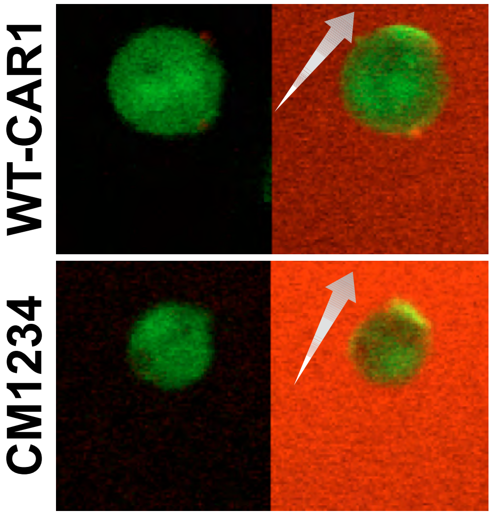

Fig. S5. PIP_3 localization in response to cAMP stimulation is similar between WT-CAR1 and CM1234 cells. Differentiated WT-CAR1 and CM1234 cells expressing a fluorescent, pleckstrin homology domain reporter [PH_{CRAC}-GFP; (Parent et al., 1998; Xu et al., 2006)] were stimulated with a point source gradient of cAMP mixed with Alexa 633 dye to determine localized PIP_3 accumulation in live cells. Cells were immobilized with 10 μM Latrunculin B to inhibit feedback from the actin cytoskeleton. Merged images are shown at 0 and 60 sec. Direction of increasing cAMP concentration is indicated with arrow.

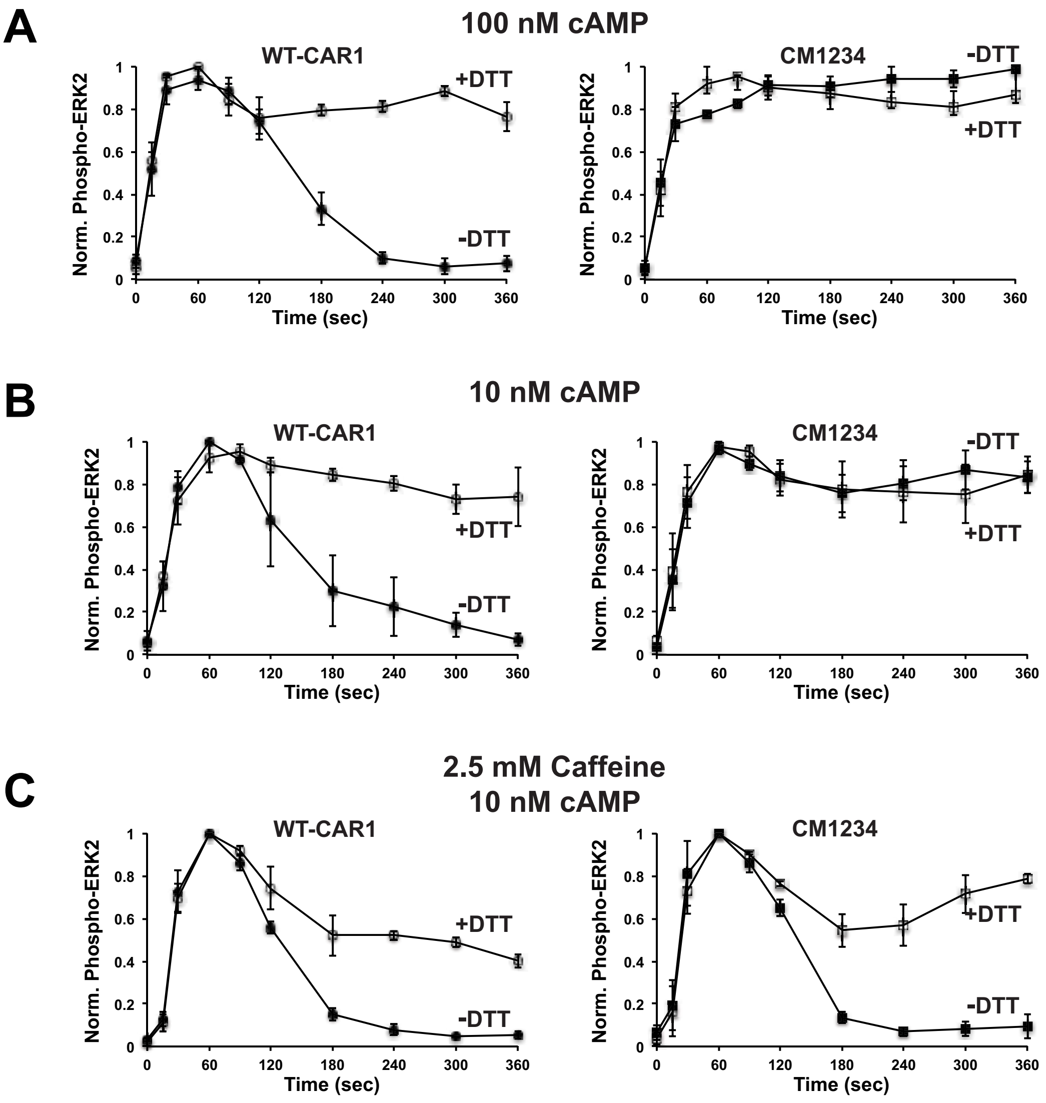

D

-DTT vs +DTT	WT-CAR1	CM1234
100 nM cAMP	<0.0001	0.39
10 nM cAMP	<0.0001	1.0
10 nM cAMP +Caffeine	<0.0001	<0.0001

WT-CAR1 vs CM1234	-DTT	+DTT
100 nM cAMP	<0.0001	0.29
10 nM cAMP	<0.0001	1.0
10 nM cAMP +Caffeine	0.98	0.08

Fig. S6. Elevated cAMP production in CM1234 cells promotes persistent ERK2 phosphorylation. A. Quantified ERK2 phosphorylation data for cells stimulated with 100 nM cAMP as in Figure 6. WT-CAR1 (n=4) and CM1234 (n=2) cells were differentiated and stimulated at 22 °C with 100 nM cAMP in the absence or presence of 10 mM DTT, an inhibitor of extracellular PDE. ERK2 phosphorylation is transient in WT-CAR1 cells in the absence of DTT, but persists if cAMP degradation is inhibited. ERK2 phosphorylation persists in CM1234 cells in the absence or presence of DTT, consistent with continuous synthesis/ accumulation of cAMP in CM1234 cells. B. Quantified ERK2 phosphorylation data from experiments in parallel to Figure 6D. WT-CAR1 (n=2) and CM1234 (n=3) cells were differentiated and stimulated at 22 °C with 10 nM cAMP. Stimulations were performed in the absence or presence of 10 mM DTT. C. Quantified ERK2 phosphorylation data from experiments in parallel to Figure 6D. WT-CAR1 (n=2) and CM1234 (n=2) cells were differentiated and stimulated at 22 °C with 10 nM cAMP in 2.5 mM caffeine, to inhibit endogenous cAMP production, and in the absence or presence of 10 mM DTT. Transient ERK2 phosphorylation is observed in both cells in the absence of DTT, consistent with the dependency of pERK2 on the presence of continuous extracellular cAMP. Inhibition of cAMP degradation by DTT maintains partial pERK during the time course. D. ANOVA was performed on pERK curves as graphed in Figure S6 A-C. Left Panel: P values are reported for pERK2 comparisons for each strain stimulated in the absence or presence of DTT. Right Panel: P values are reported for pERK2 comparisons of WT-CAR1 and CM1234 cells for the indicated conditions. $P < 0.05$ was considered significant.

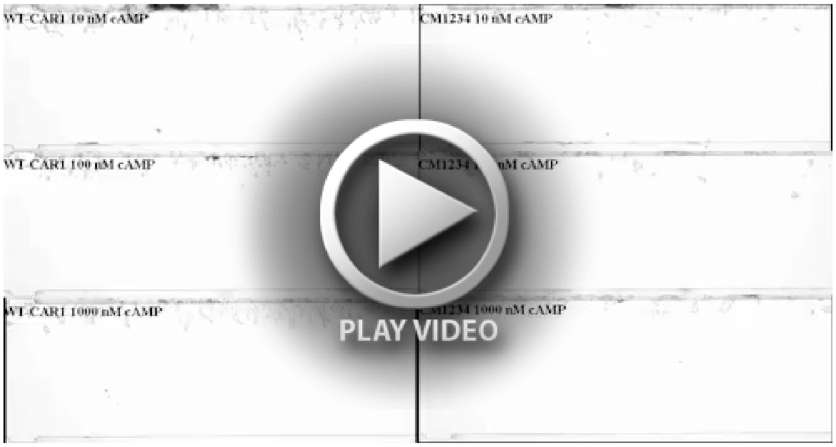

Movie 1. EZ-TAXIScan cAMP chemotaxis assay. Differentiated WT-CAR1 or CM1234 cells were exposed to linear gradients established with the indicated concentrations of cAMP in an EZ-TAXIScan chamber (see Figure 1A). All conditions were performed simultaneously in a six well chamber. Images were captured every 15 sec and montaged into one movie. Playback is 20 frames per sec.

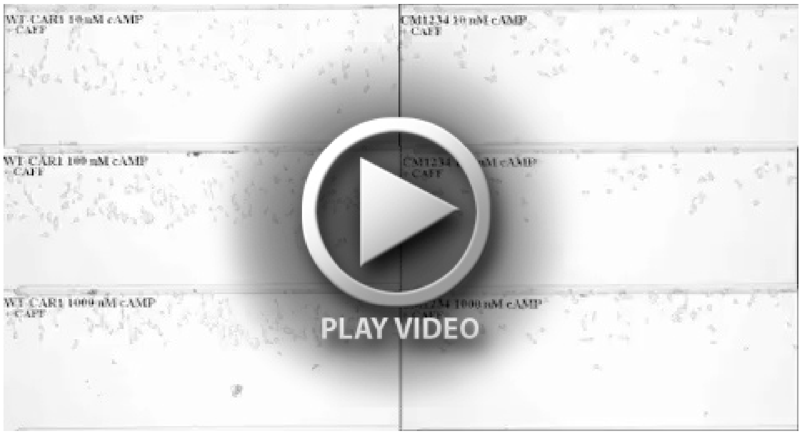

Movie 2. EZ-TAXIScan cAMP chemotaxis assay in the presence of 0.5 mM caffeine. Differentiated WT-CAR1 or CM1234 cells were exposed to linear gradients established with the indicated concentrations of cAMP in the presence of 0.5 mM caffeine in an EZ-TAXIScan chamber (see Figure 1B). All conditions were performed simultaneously in a six well chamber. Images were captured every 15 sec and montaged into one movie. Playback is 20 frames per sec.

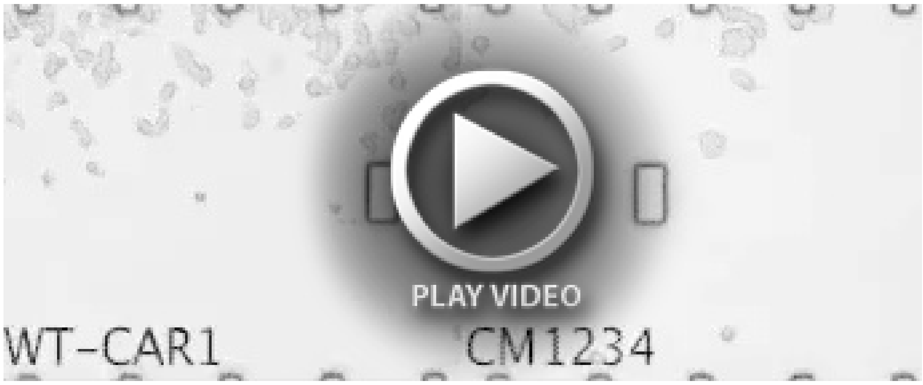

Movie 3. EZ-TAXIScan chemotaxis assay of WT-CAR1 and CM1234 cells in the presence of 2 mM caffeine. Differentiated WT-CAR1 or CM1234 cells were exposed to linear gradients established with the indicated concentrations of cAMP in the presence of 2 mM caffeine in an EZ-TAXIScan chamber. Images were captured every 15 sec and montaged into one movie. Playback is 20 frames per sec.

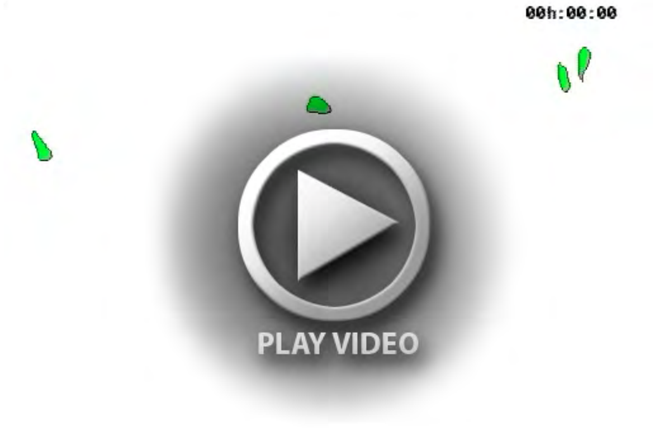

Movie 4. Selected WT-CAR1 cells in the presence of 0.5 mM caffeine traced using the DIAS software package are shown. Cells were exposed to a linear gradient established with 100 nM cAMP in the presence of 0.5 mM caffeine (see Figure 1C). Gray indicates the cell's shape in the current frame, green indicates the area (pseudopodia) that extends from the cell in the next frame, and red indicates the area (uropod) that trails in previous frame. Images were captured every 15 sec. Playback is 5 frames per sec.

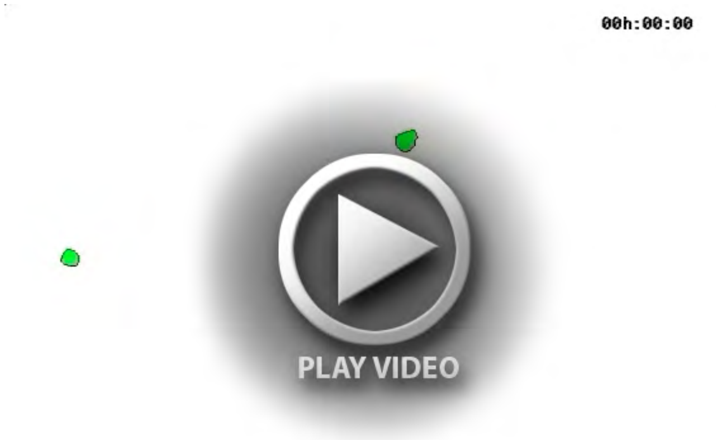

Movie 5. Selected CM1234 cells in the presence of 0.5 mM caffeine traced using the DIAS software package are shown. Cells were exposed to a linear gradient established with 100 nM cAMP in the presence of 0.5 mM caffeine (see Figure 1C). See Movie 4 legend for description of colors. Images were captured every 15 sec. Playback is 5 frames per sec.

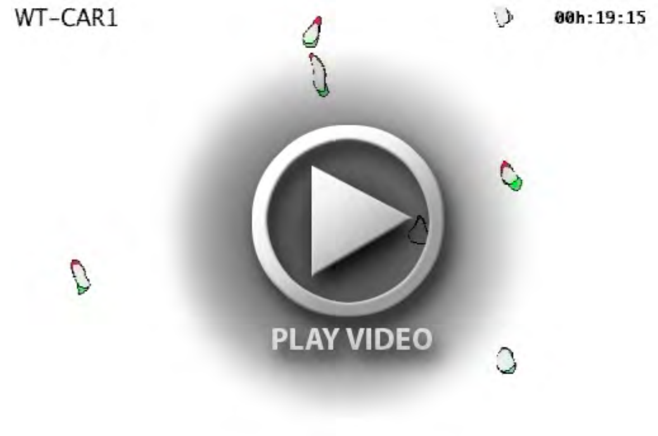

Movie 6. Selected WT-CAR1 cells at low density in the absence of caffeine traced using the DIAS software package are shown. Cells were exposed to a linear gradient established with 1000 nM cAMP in the absence of caffeine. See Movie 4 legend for description of colors. Images were captured every 15 sec. Playback is 10 frames per sec.

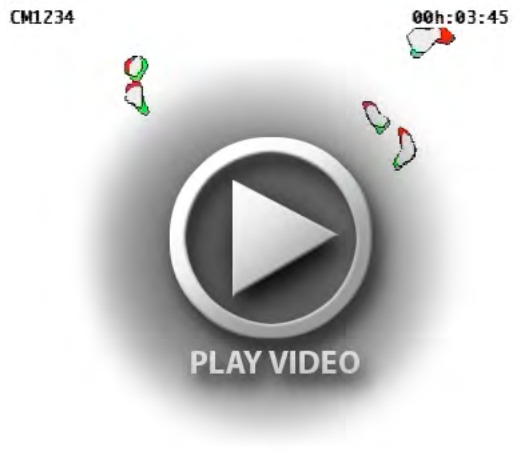

Movie 7. Selected CM1234 cells at low density in the absence of caffeine traced using the DIAS software package are shown. Cells were exposed to a linear gradient established with 1000 nM cAMP in the absence of caffeine. See Movie 4 legend for description of colors. Images were captured every 15 sec. Playback is 10 frames per sec.

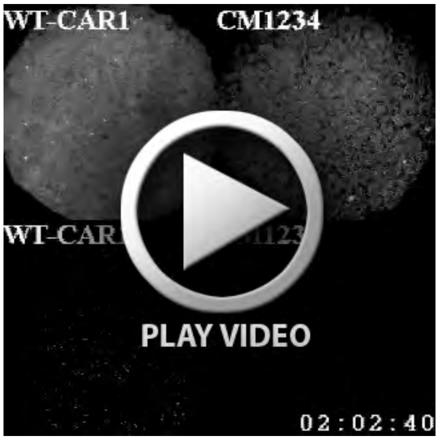

Movie 8. Development on agarose imaged by darkfield microscopy. WT-CAR1 and CM1234 cells were starved on non-nutrient agarose surfaces and oscillatory cell shape changes due to cAMP relay were imaged by darkfield microscopy. Top panels show the raw darkfield sequence. Bottom panels show the enhanced frame-subtracted sequence. Playback is 20 frames per second. See Figure 2 for details. Time is indicated in hr:min:sec, with major differences observed by 4 hrs.

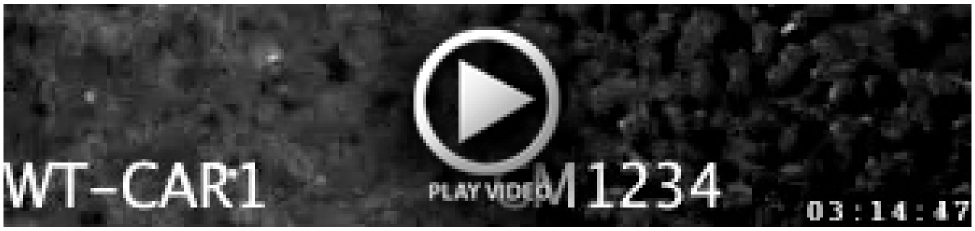

Movie 9. Zoom of Movie 8. A subregion of the raw image sequence shown in Movie 8 is displayed with a 2x zoom to show the absence of head-to-tail streams in CM1234 cells. Time is indicated in hr:min:sec. Playback is 20 frames per second.

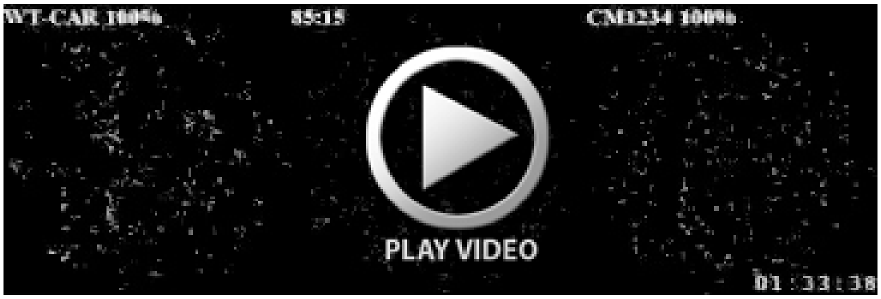

Movie 10. Darkfield wave propagation is disrupted by a small percentage of CM1234 cell in chimeras. WT-CAR1, CM1234, or a chimera consisting of 85% WT-CAR1 cells and 15% CM1234 cells (middle image) were starved on non-nutrient agarose surfaces and oscillatory cell shape changes due to cAMP relay were imaged by darkfield microscopy and digitally recorded (see Figure 3). Shown is the frame-subtracted image sequence. Time is indicated in hr:min:sec with major differences starting by 2 hrs. Note that cells were plated ~2hr prior to the start of recording to accommodate later developmental structures. Playback is 20 frames per second.

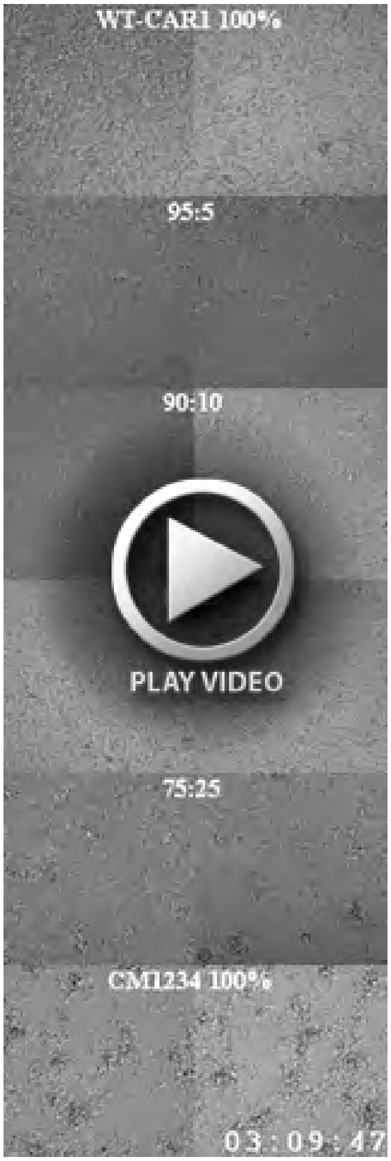

Movie 11. WT-CAR1 cell-to-cell streaming is disrupted and mound size decreased by a small percentage of CM1234 cells during chimeric development. WT-CAR1, CM1234 cells, or chimeras consisting of the indicated ratios of WT-CAR1 and CM1234 cells were starved under buffer on plastic surfaces in a multiwell plate and imaged by time lapse microscopy (see Figure 3). Two examples for each ratio are shown. Playback is 10 frames per second. Time is indicated in hr:min:sec, with major differences observed by 4 hrs.

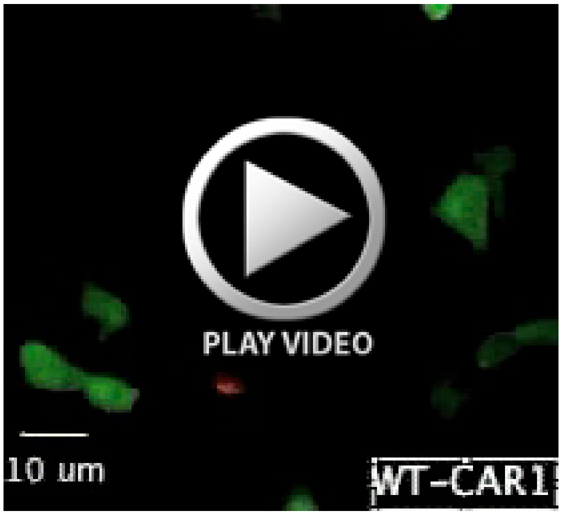

Movie 12. PIP_3 dynamics in WT-CAR1 cells migrating toward a micropipette. Shown are WT-CAR1 cells expressing the PIP_3 reporter PH_{CRAC}-GFP imaged by confocal microscopy at ~1.5 sec intervals using a 40x objective lens with a zoom factor of 2. The micropipette was filled with 1 mM cAMP and Alexa 633 and was moved to show changes in PIP_3 localization.

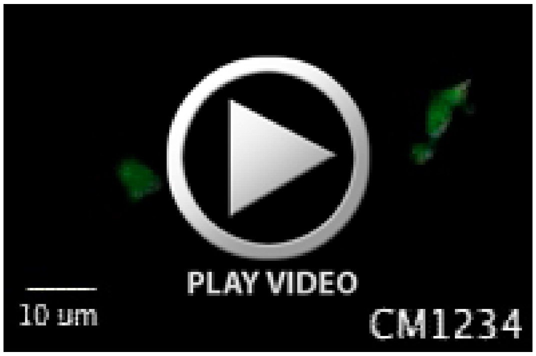

Movie 13. PIP_3 dynamics in CM1234 cells migrating toward a micropipette. Shown are CM1234 cells expressing the PIP_3 reporter PH_{CRAC}-GFP imaged by confocal microscopy at ~1.5 sec intervals using a 40x objective lens with a zoom factor of. The micropipette was filled with 1 μM cAMP and Alexa 633 and was moved to show changes in PIP_3 localization.

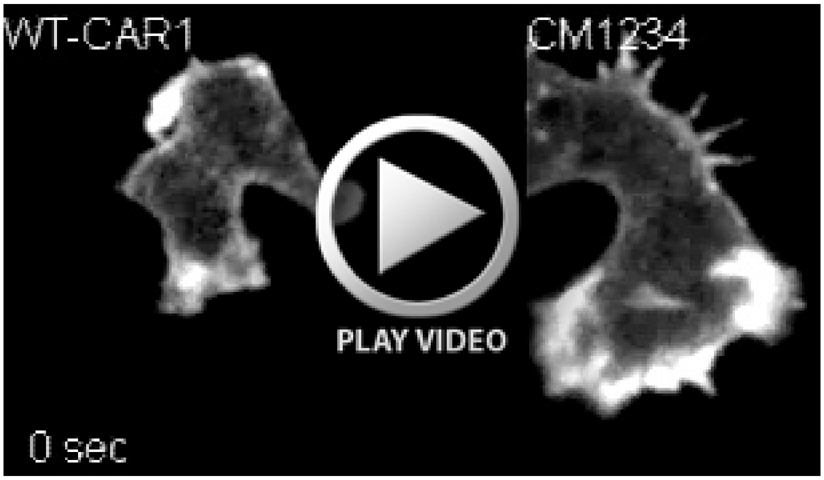

Movie 14. Actin dynamics in WT-CAR1 and CM1234 cells captured by spinning disk confocal microscopy. Shown are maximum projections of WT-CAR1 and CM1234 cells expressing the F-actin probe ABD-GFP imaged at 2 sec intervals using a 100x objective lens by spinning disk confocal microscopy. The "Primary" response to a 1 μM cAMP stimulus (applied on previous frame) is indicated. CM1234 cells maintain an elevated secondary response throughout the movie.

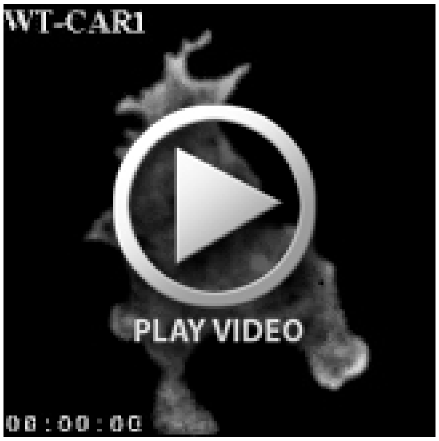

Movie 15. Actin dynamics in WT-CAR1 cells captured by TIR-FM. Shown are two WT-CAR1 cells expressing the F-actin probe ABD-GFP imaged at 2 sec intervals using a 150x objective lens by TIR-FM to capture F-actin dynamics proximal to the cover slip (see Figure 7). The points of "Stimulation" with 1 μM cAMP, the transient increase of F-actin to the cell periphery, "Primary," and the "Secondary" response are indicated.

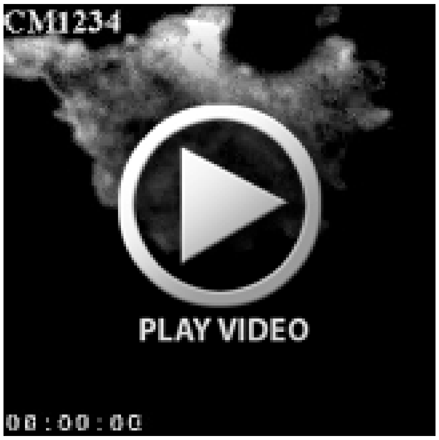

Movie 16. Actin dynamics in CM1234 cells captured by TIR-FM. Shown are two CM1234 cells expressing the F-actin probe ABD-GFP imaged at 2 sec intervals using a 150x objective lens by TIR-FM to capture F-actin dynamics proximal to the cover slip (see Figure 7). The points of "Stimulation" with 1 μM cAMP, the transient increase of F-actin to the cell periphery, "Primary," and the "Secondary" response are indicated.
